# Supplementary figures and images for: Tribological Properties of Nitrate Graphite Foils
Source: Nanomaterials (Basel). 2024 Sep 15;14(18):1499. doi: 10.3390/nano14181499 (PMC11434555; doi:10.3390/nano14181499)

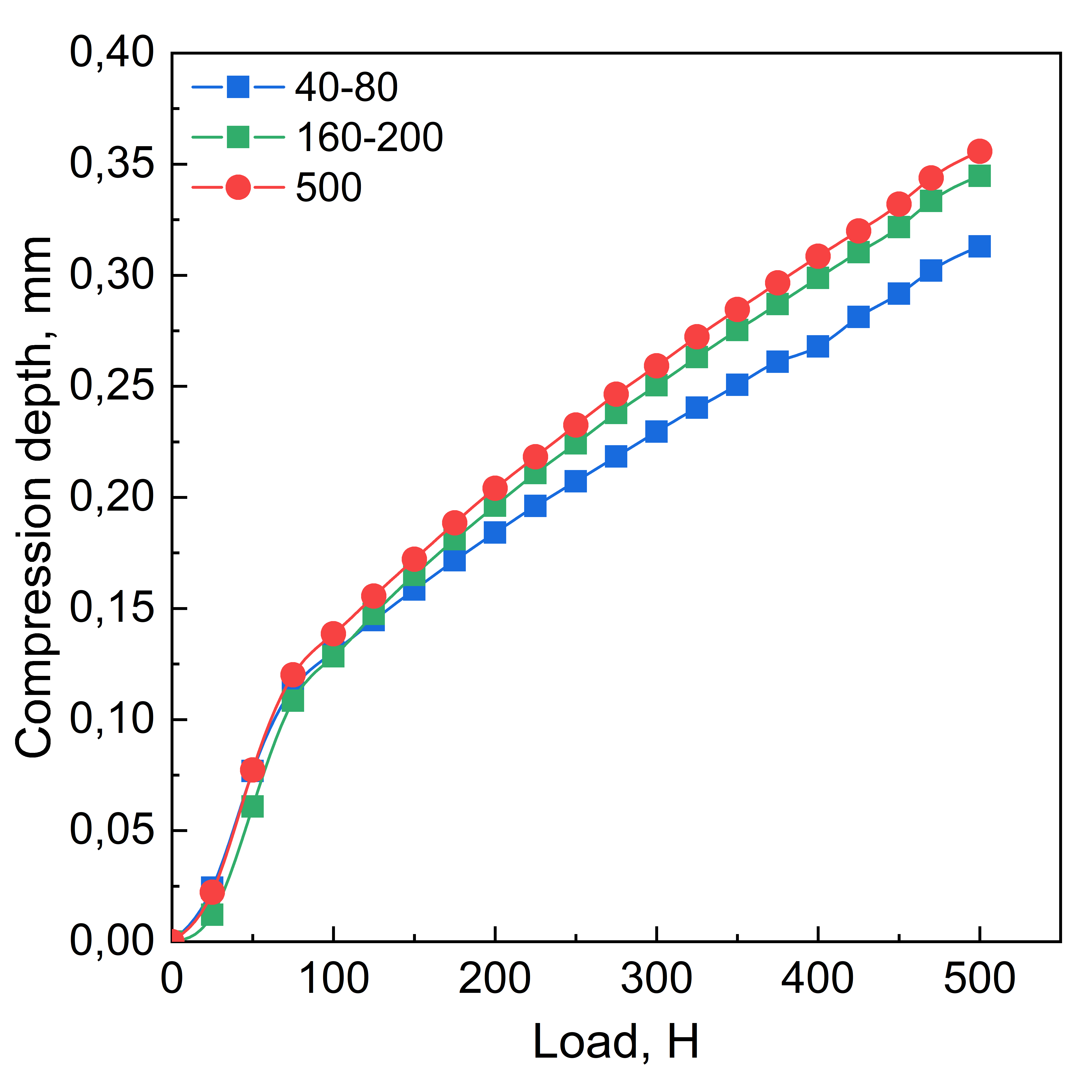

Supplement: Supplementary file 1 [file nanomaterials-14-01499-s001.zip › Hardness_done/compression depth from load.png]

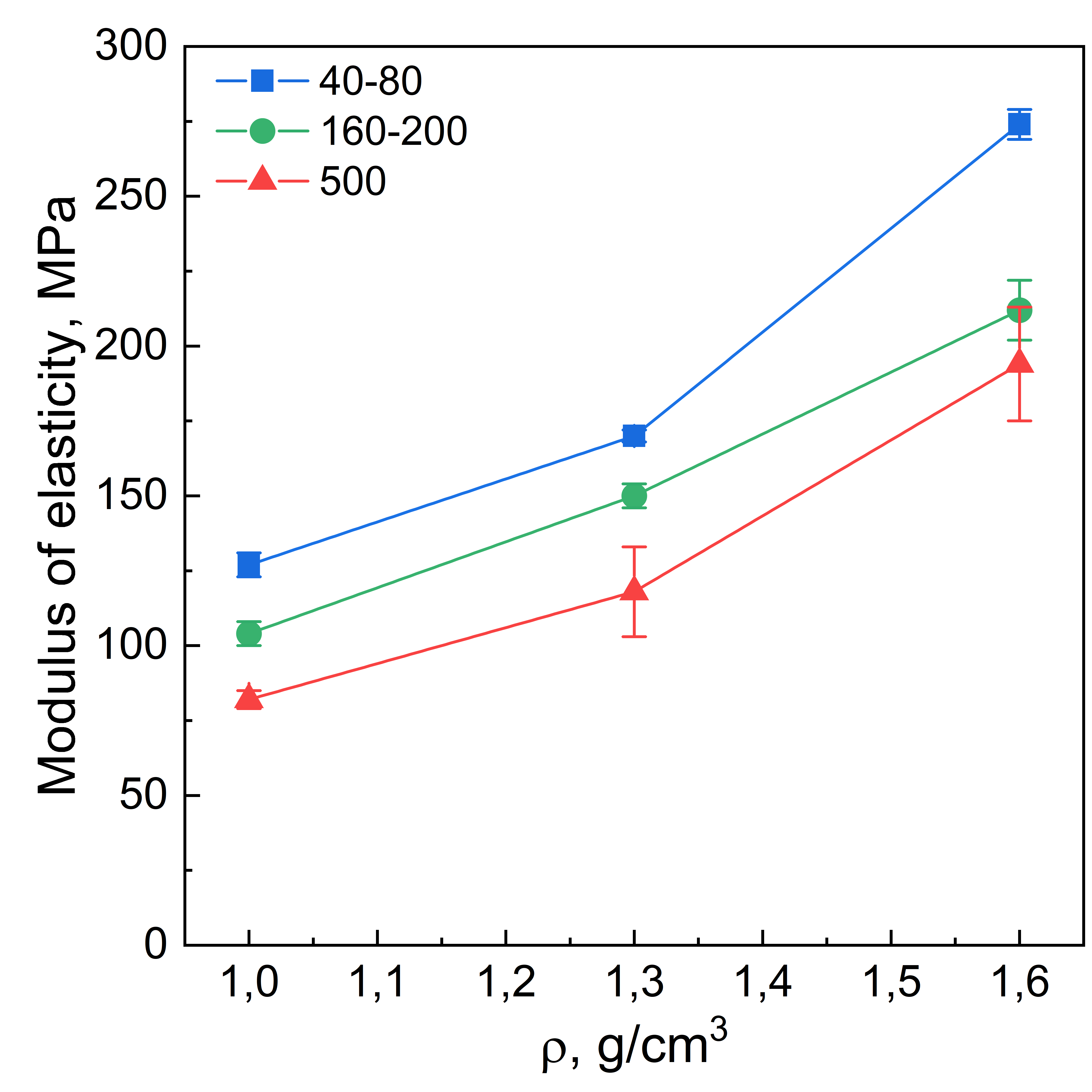

Supplement: Supplementary file 1 [file nanomaterials-14-01499-s001.zip › Hardness_done/elastic modules.png]

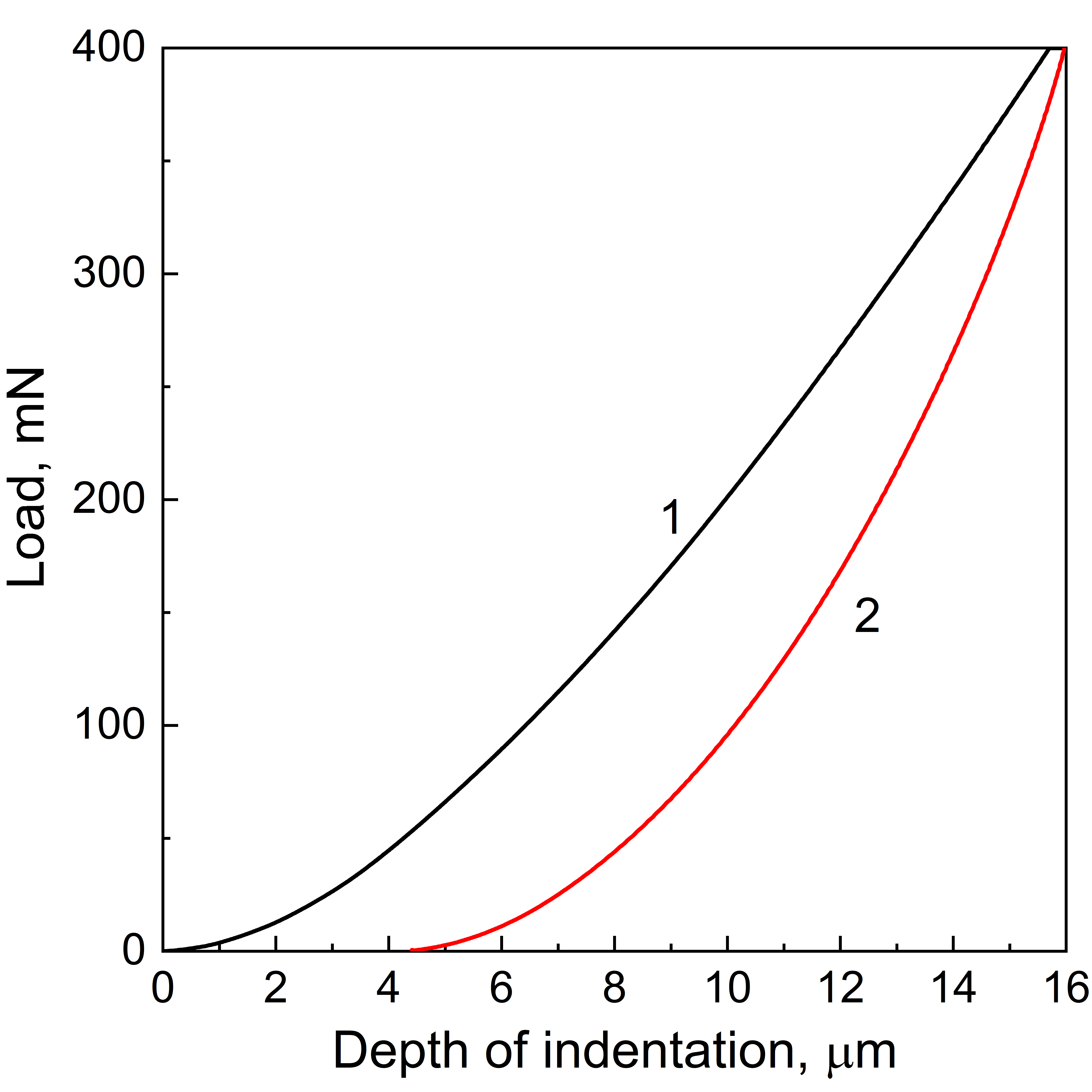

Supplement: Supplementary file 1 [file nanomaterials-14-01499-s001.zip › Hardness_done/load-unload.png]

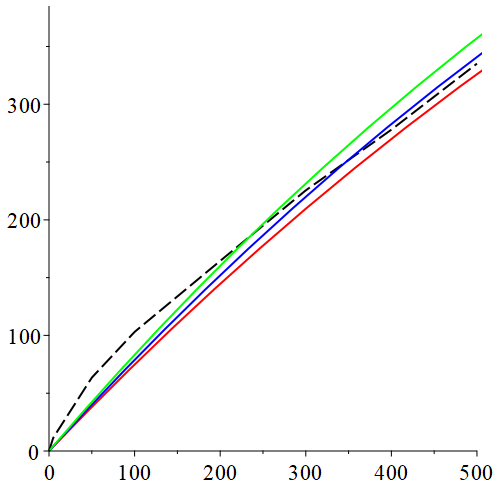

Supplement: Supplementary file 1 [file nanomaterials-14-01499-s001.zip › Model/dependences of the vertical displacement of the sample on the load/hyper-elasticity_v01.png]

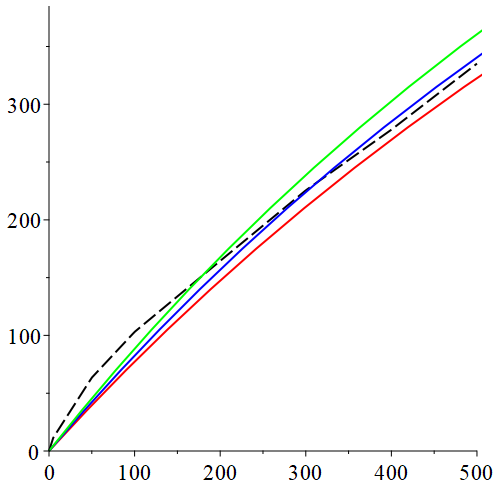

Supplement: Supplementary file 1 [file nanomaterials-14-01499-s001.zip › Model/dependences of the vertical displacement of the sample on the load/hyper-elasticity_v03.png]

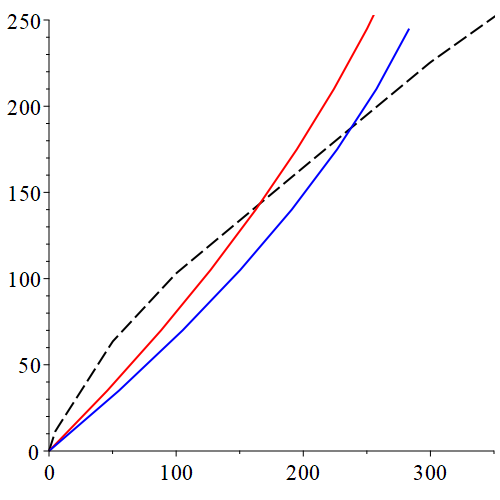

Supplement: Supplementary file 1 [file nanomaterials-14-01499-s001.zip › Model/dependences of the vertical displacement of the sample on the load/linear elasticity_E10.png]

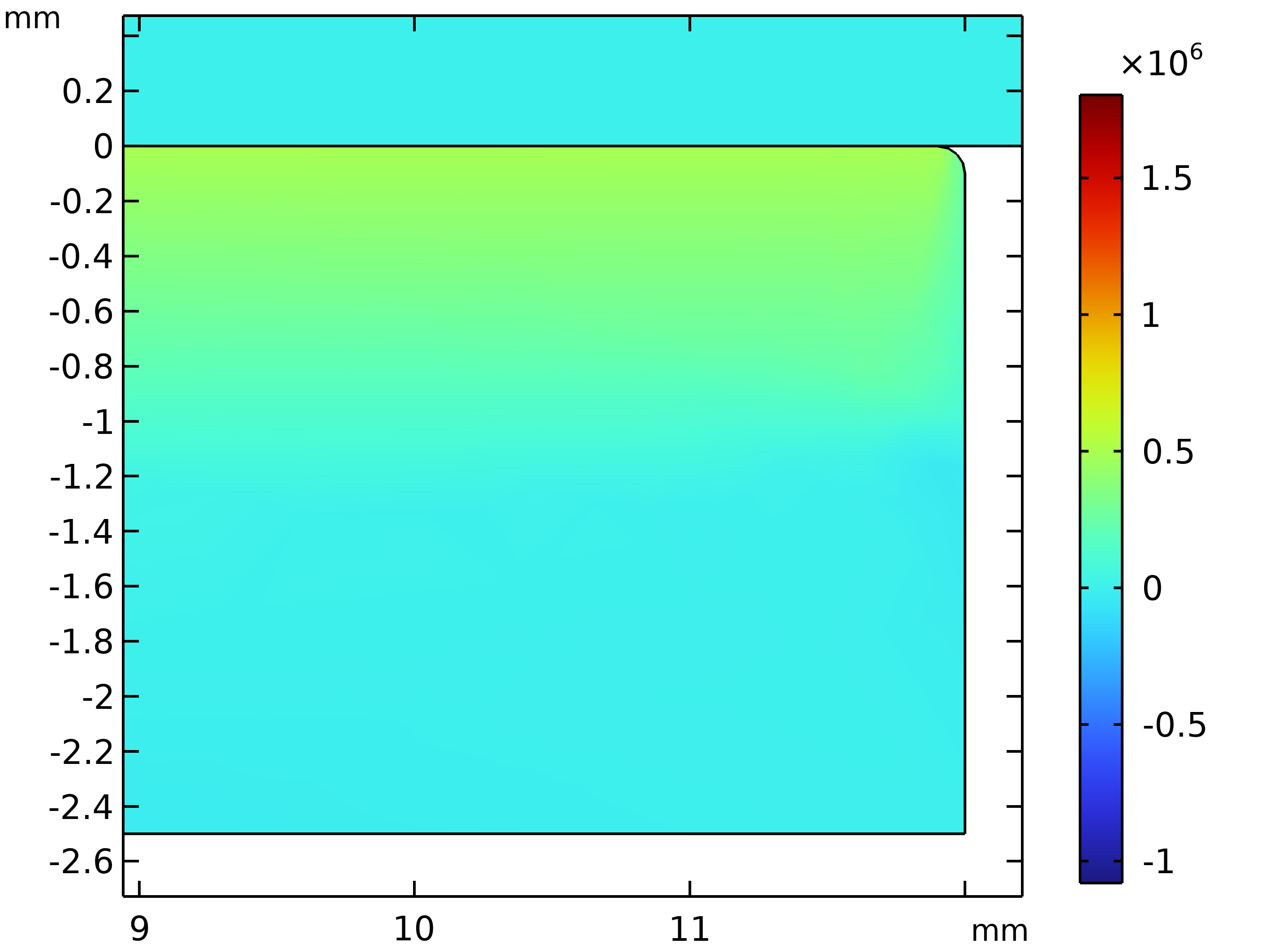

Supplement: Supplementary file 1 [file nanomaterials-14-01499-s001.zip › Model/distribution of the stress tensor component a╙_yz/anisotropic material.png]

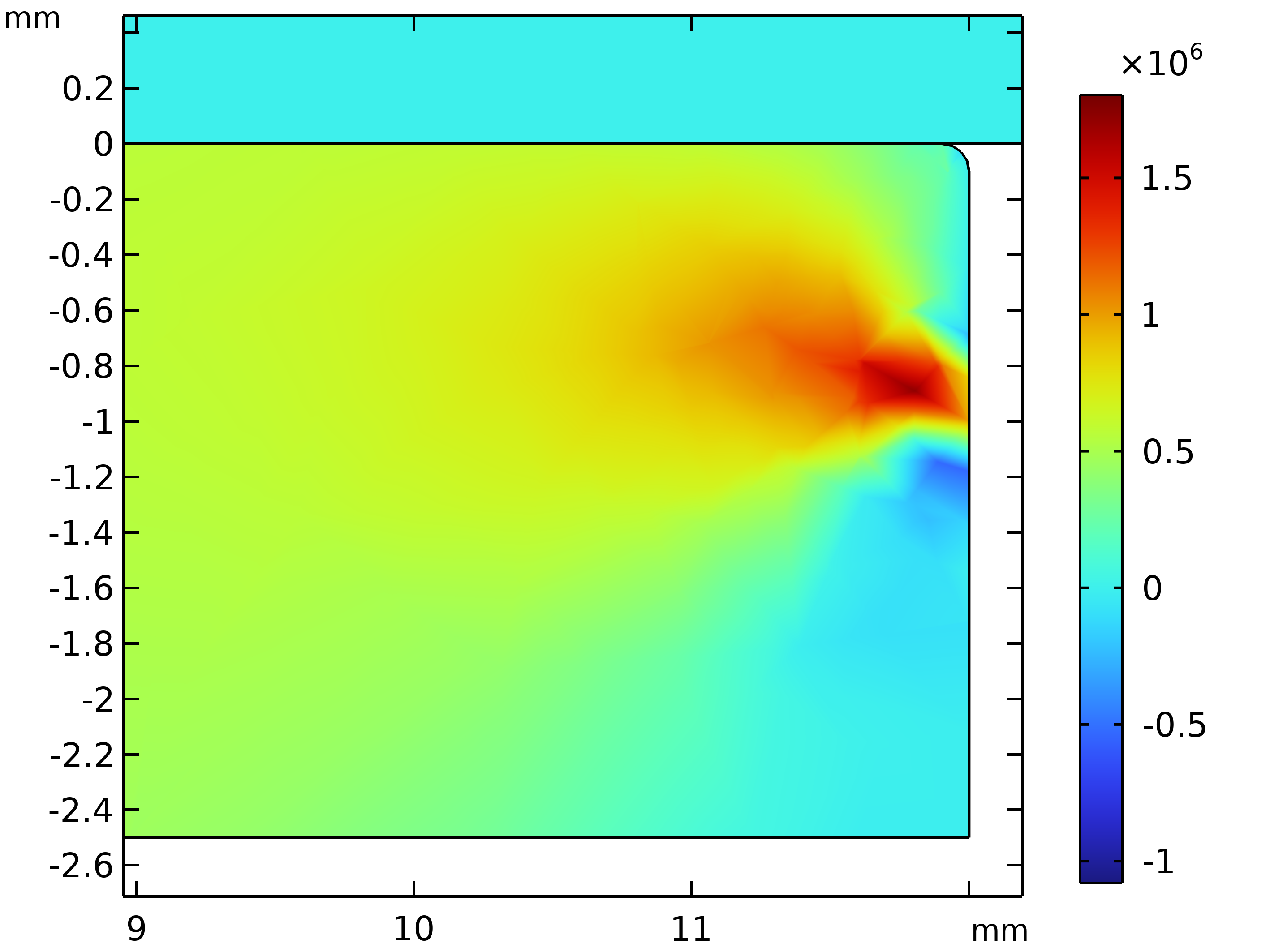

Supplement: Supplementary file 1 [file nanomaterials-14-01499-s001.zip › Model/distribution of the stress tensor component a╙_yz/isotropic material.png]

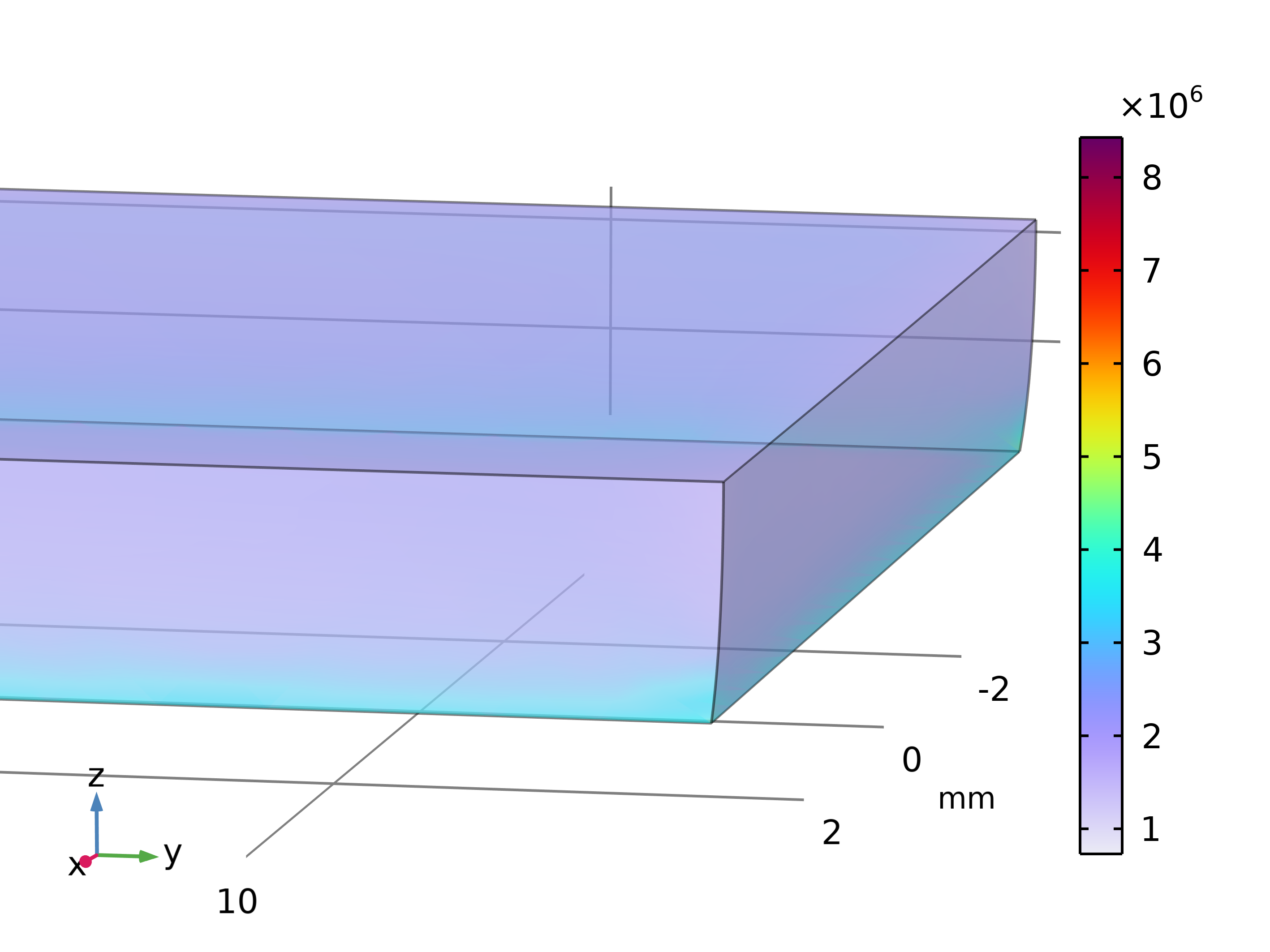

Supplement: Supplementary file 1 [file nanomaterials-14-01499-s001.zip › Model/von Mises stress distribution/a═=0.1.png]

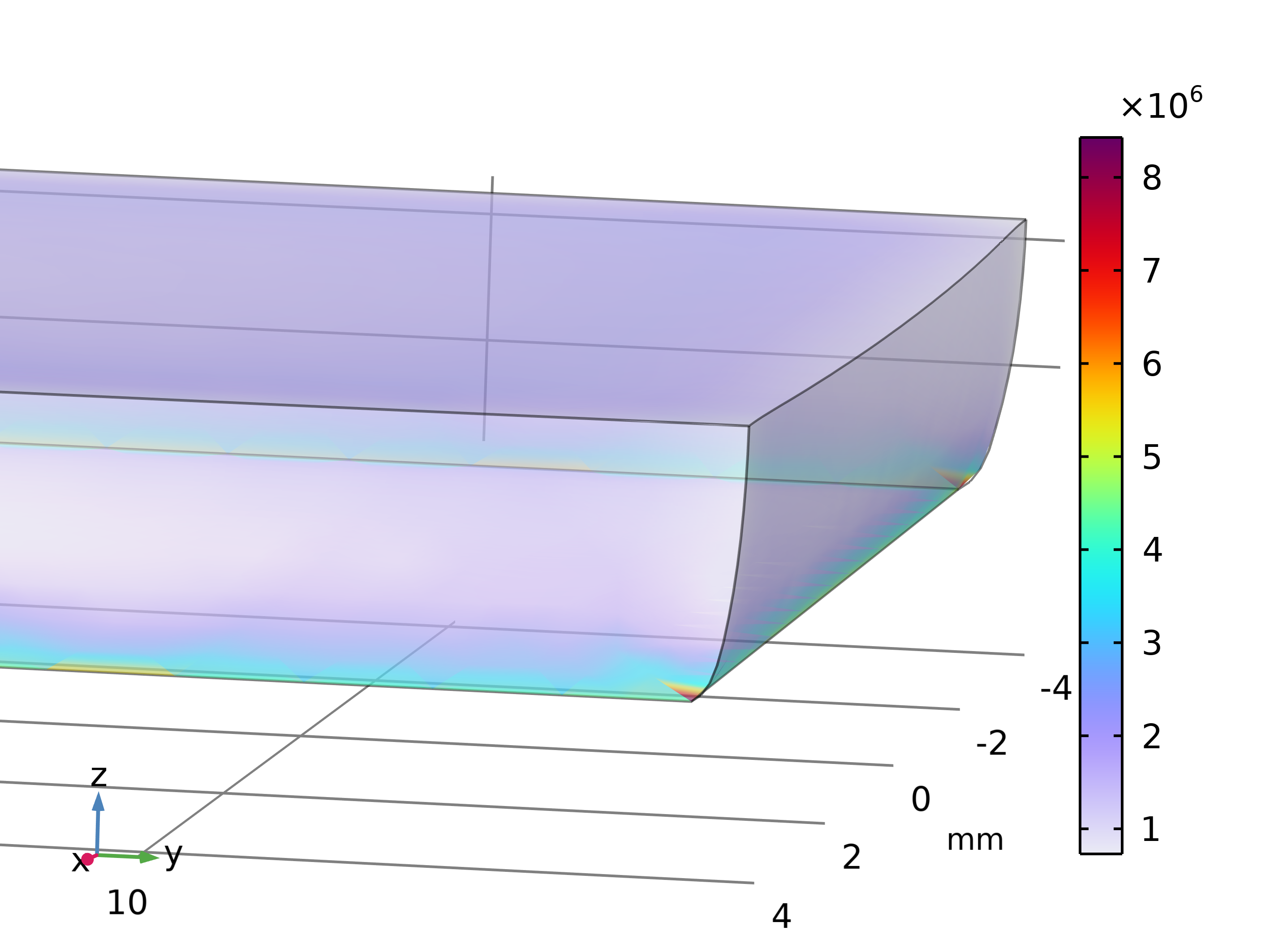

Supplement: Supplementary file 1 [file nanomaterials-14-01499-s001.zip › Model/von Mises stress distribution/a═=0.3.png]

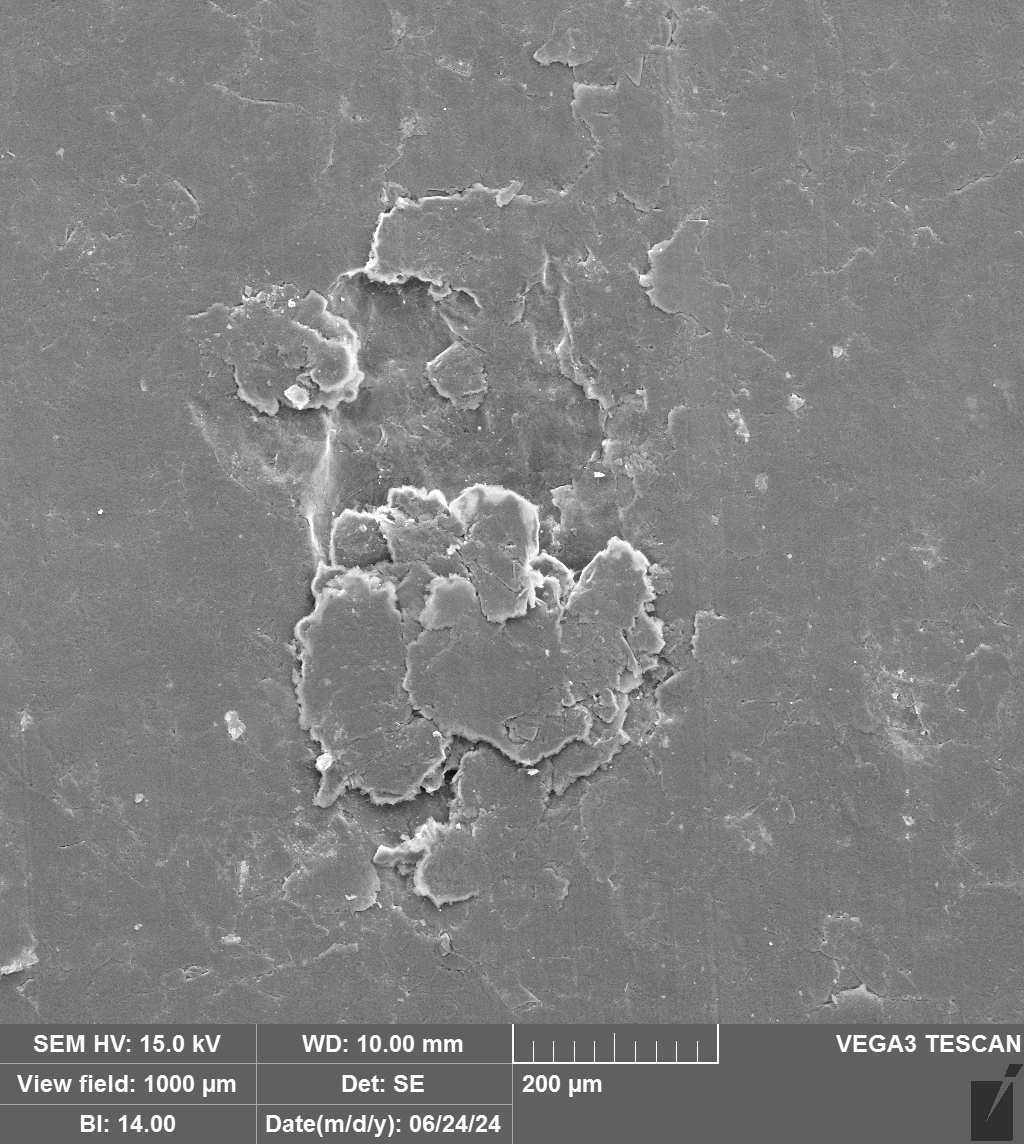

Supplement: Supplementary file 1 [file nanomaterials-14-01499-s001.zip › SEM/0001_40-80/1_1000.tif]

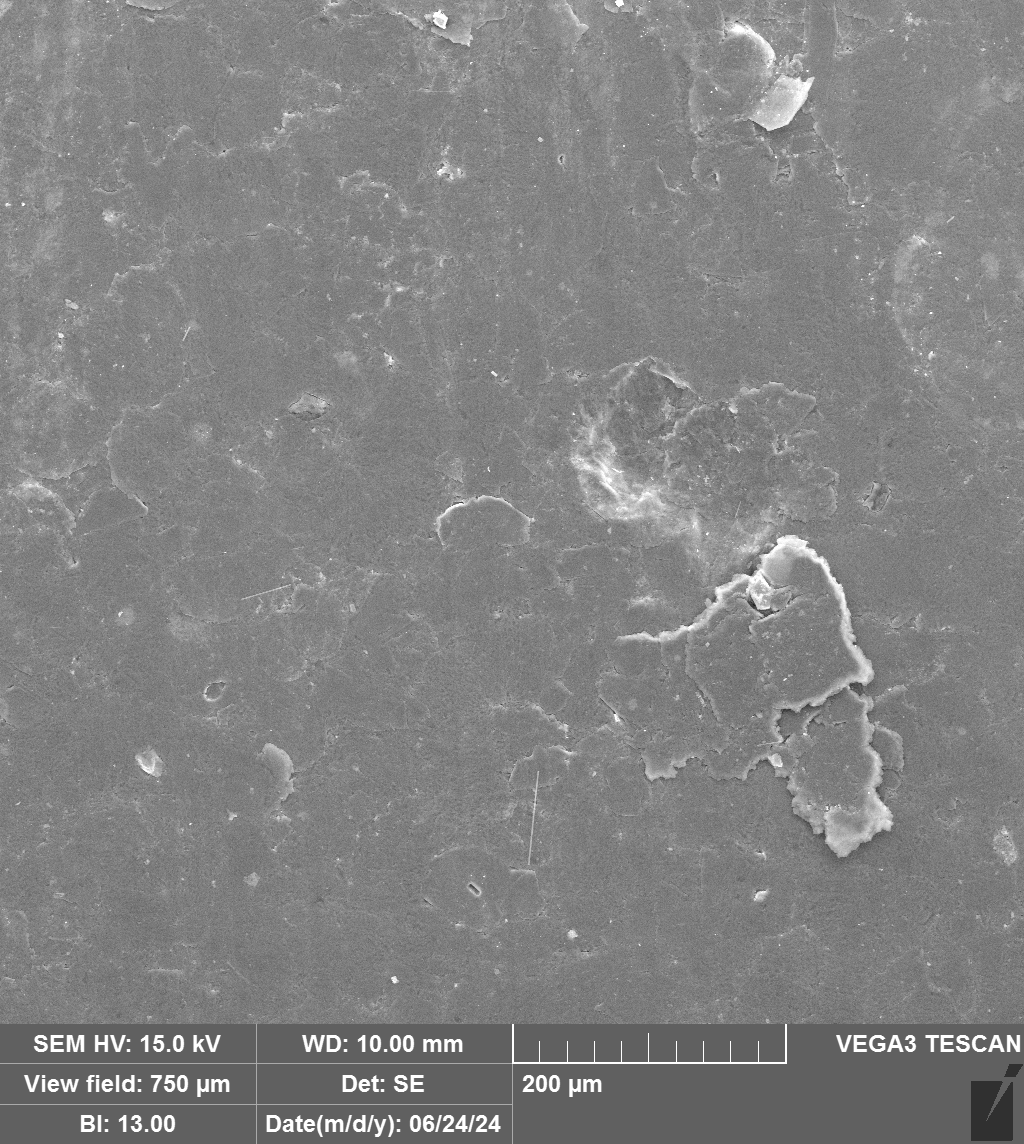

Supplement: Supplementary file 1 [file nanomaterials-14-01499-s001.zip › SEM/0001_40-80/1_750.tif]

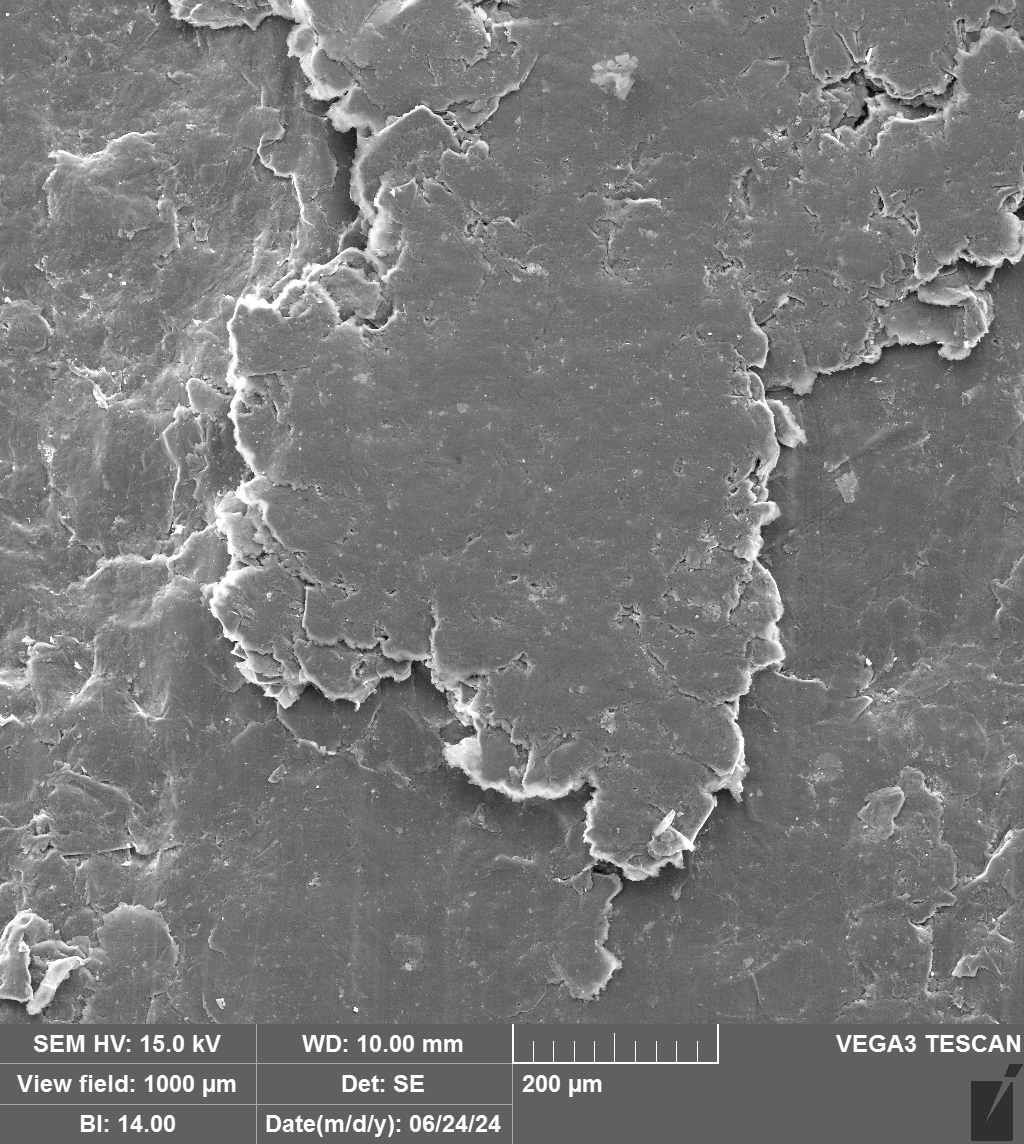

Supplement: Supplementary file 1 [file nanomaterials-14-01499-s001.zip › SEM/01Hz_40-80/1_1000.tif]

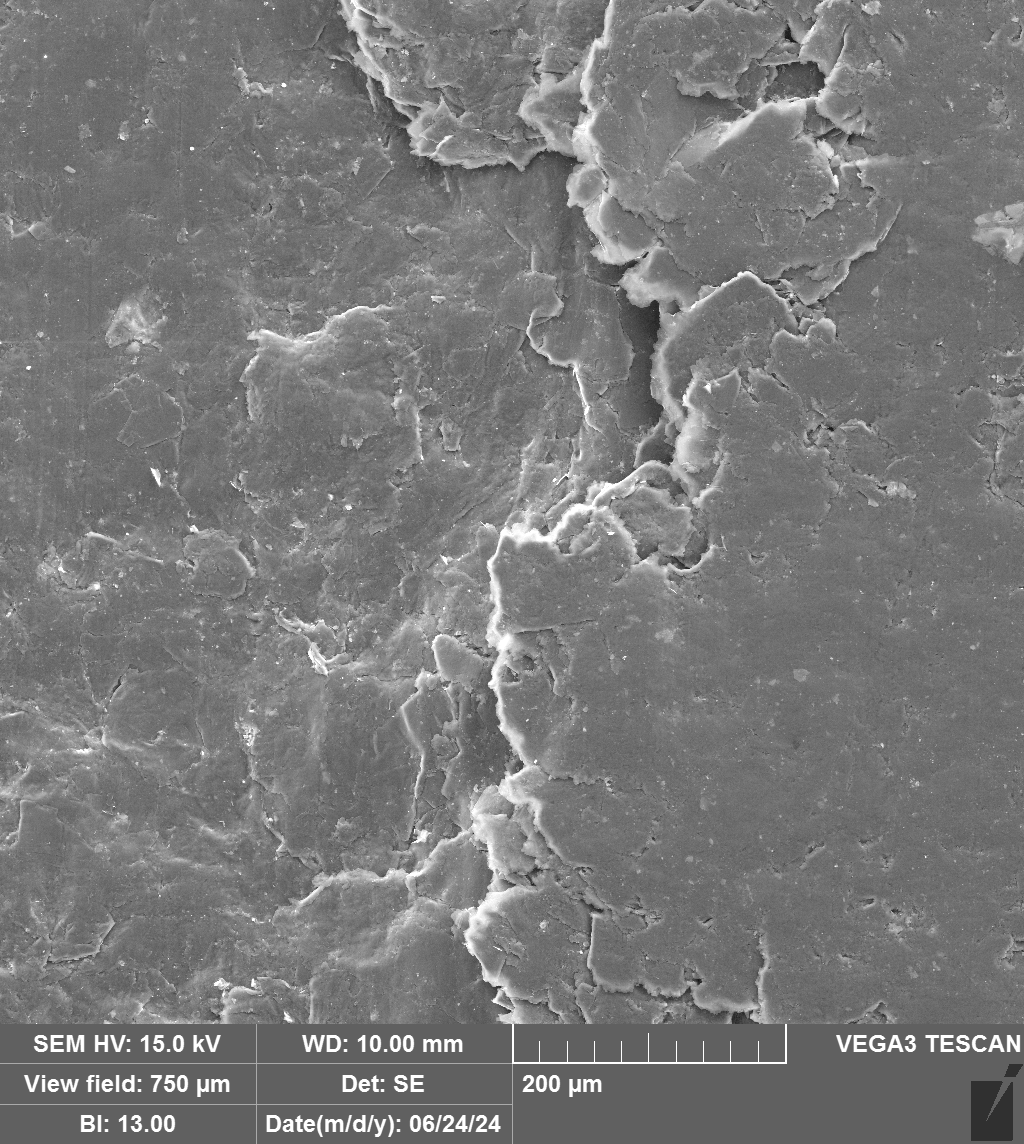

Supplement: Supplementary file 1 [file nanomaterials-14-01499-s001.zip › SEM/01Hz_40-80/1_750.tif]

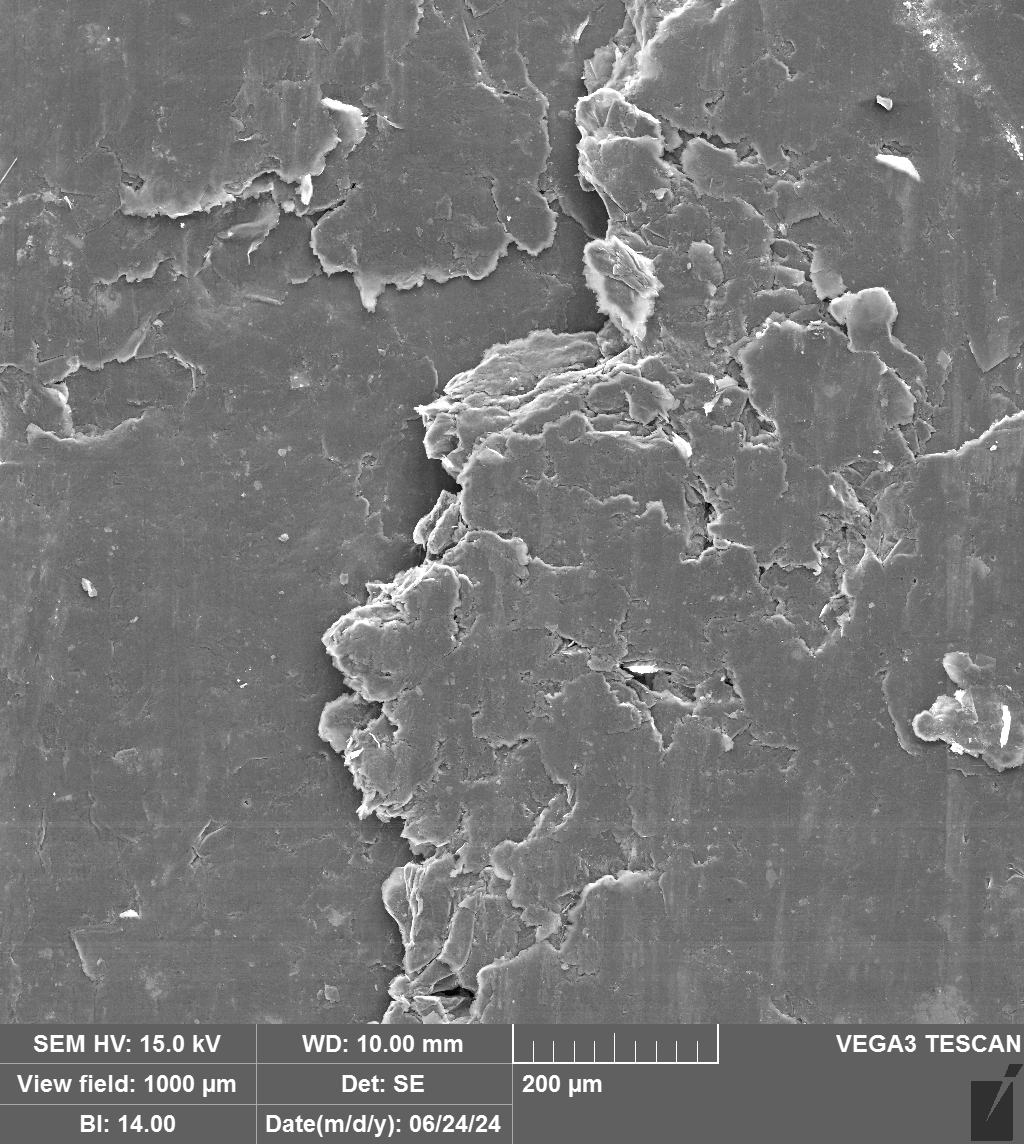

Supplement: Supplementary file 1 [file nanomaterials-14-01499-s001.zip › SEM/1Hz_40-80/1_1000.tif]

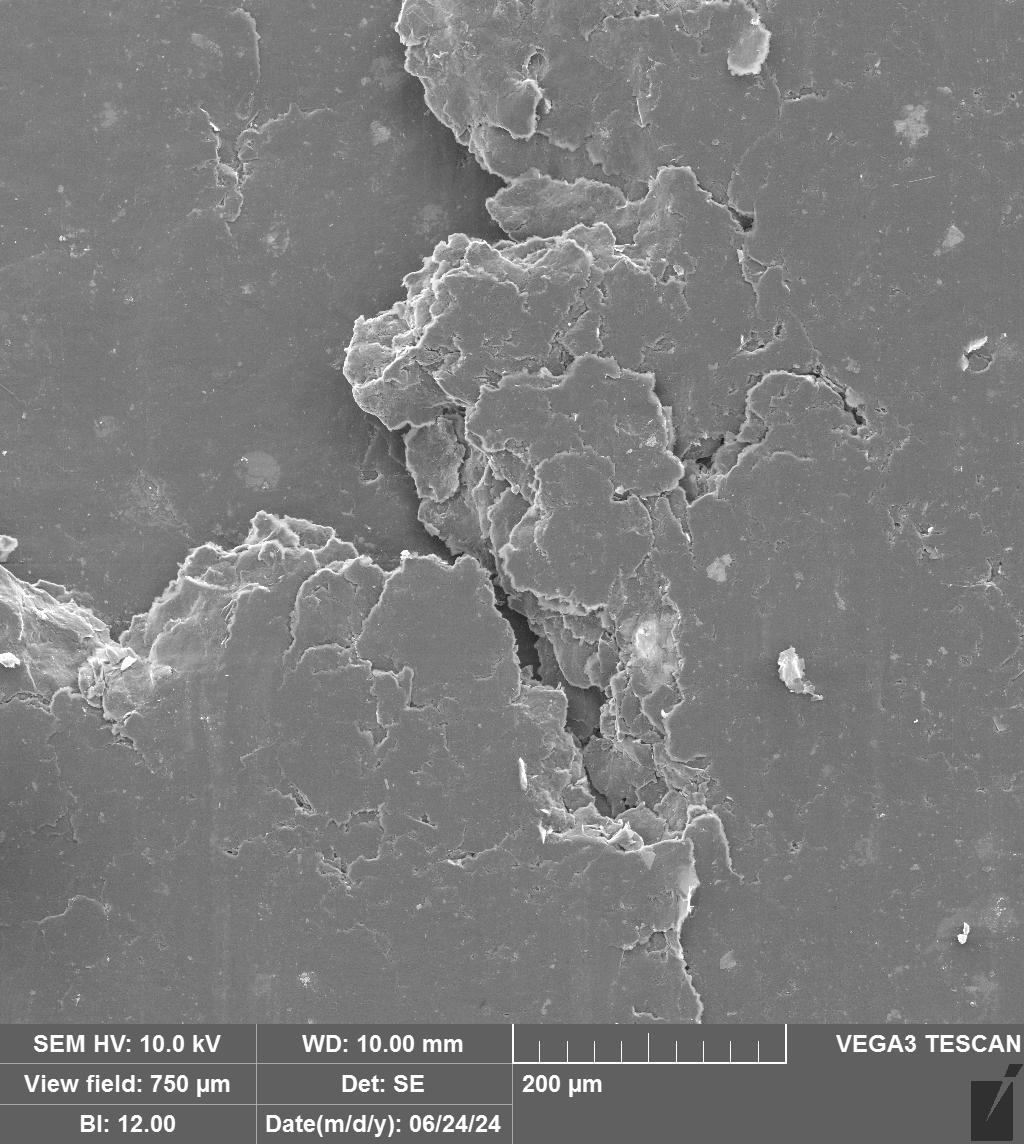

Supplement: Supplementary file 1 [file nanomaterials-14-01499-s001.zip › SEM/1Hz_40-80/1_750.tif]

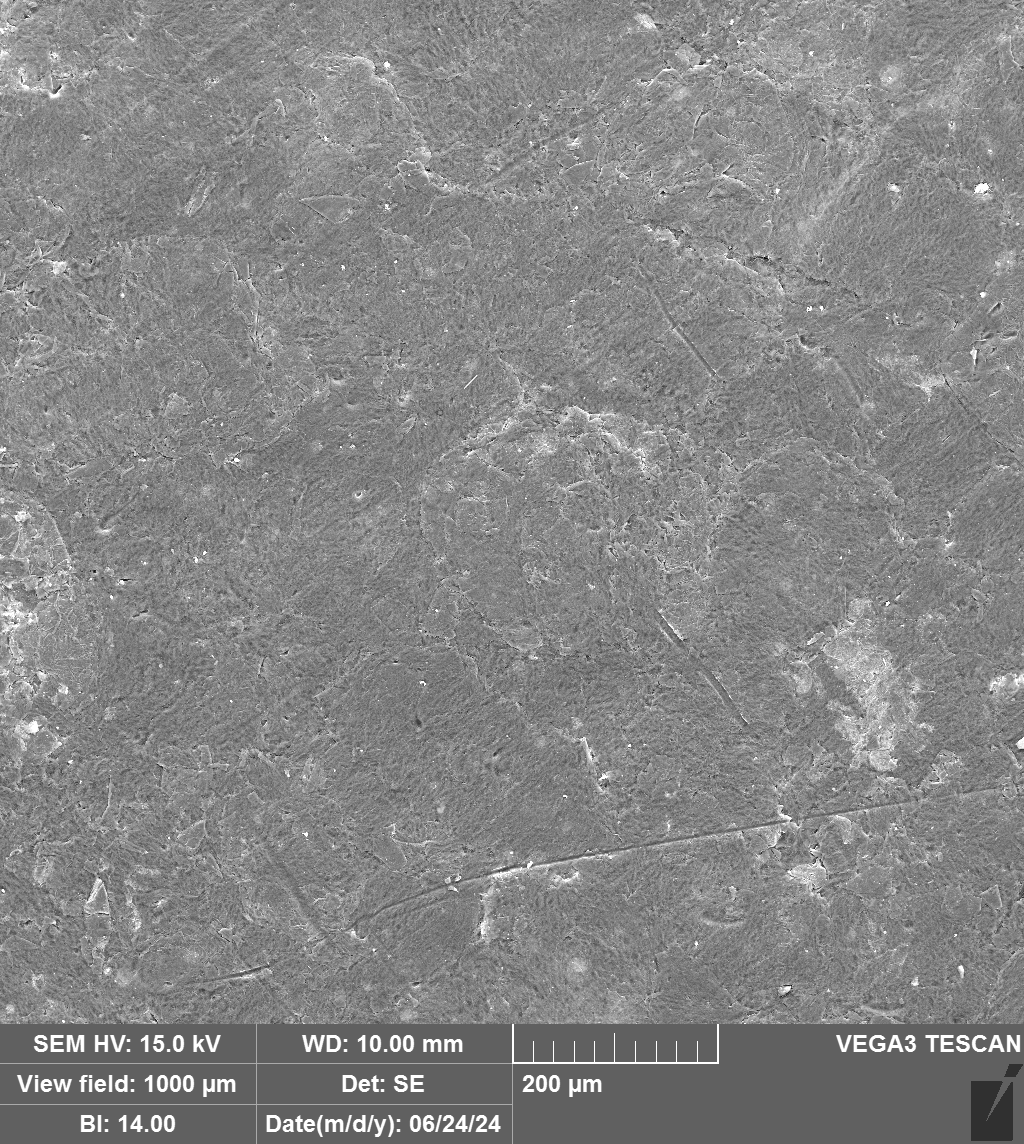

Supplement: Supplementary file 1 [file nanomaterials-14-01499-s001.zip › SEM/basic_40-80/1_1000.tif]

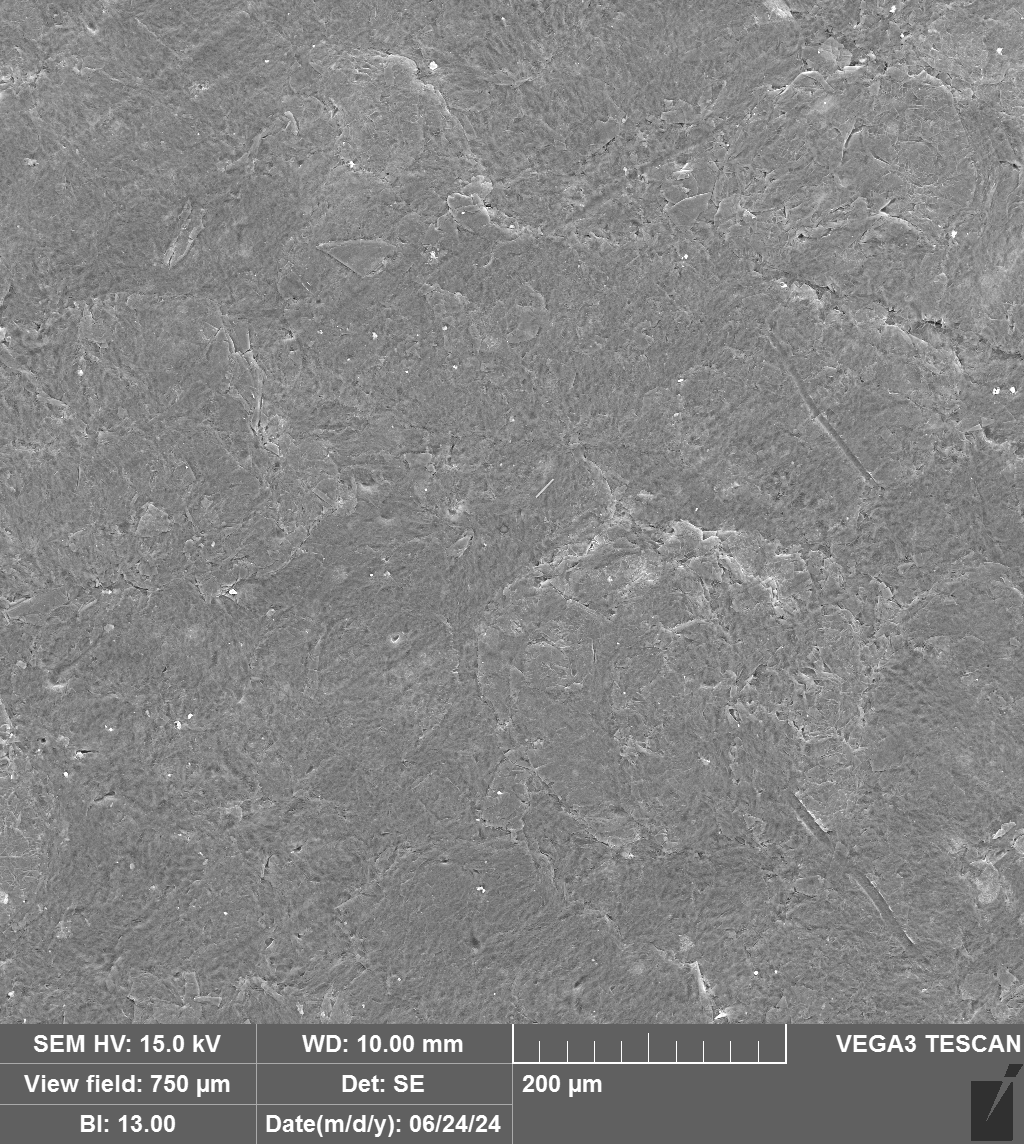

Supplement: Supplementary file 1 [file nanomaterials-14-01499-s001.zip › SEM/basic_40-80/1_750.tif]

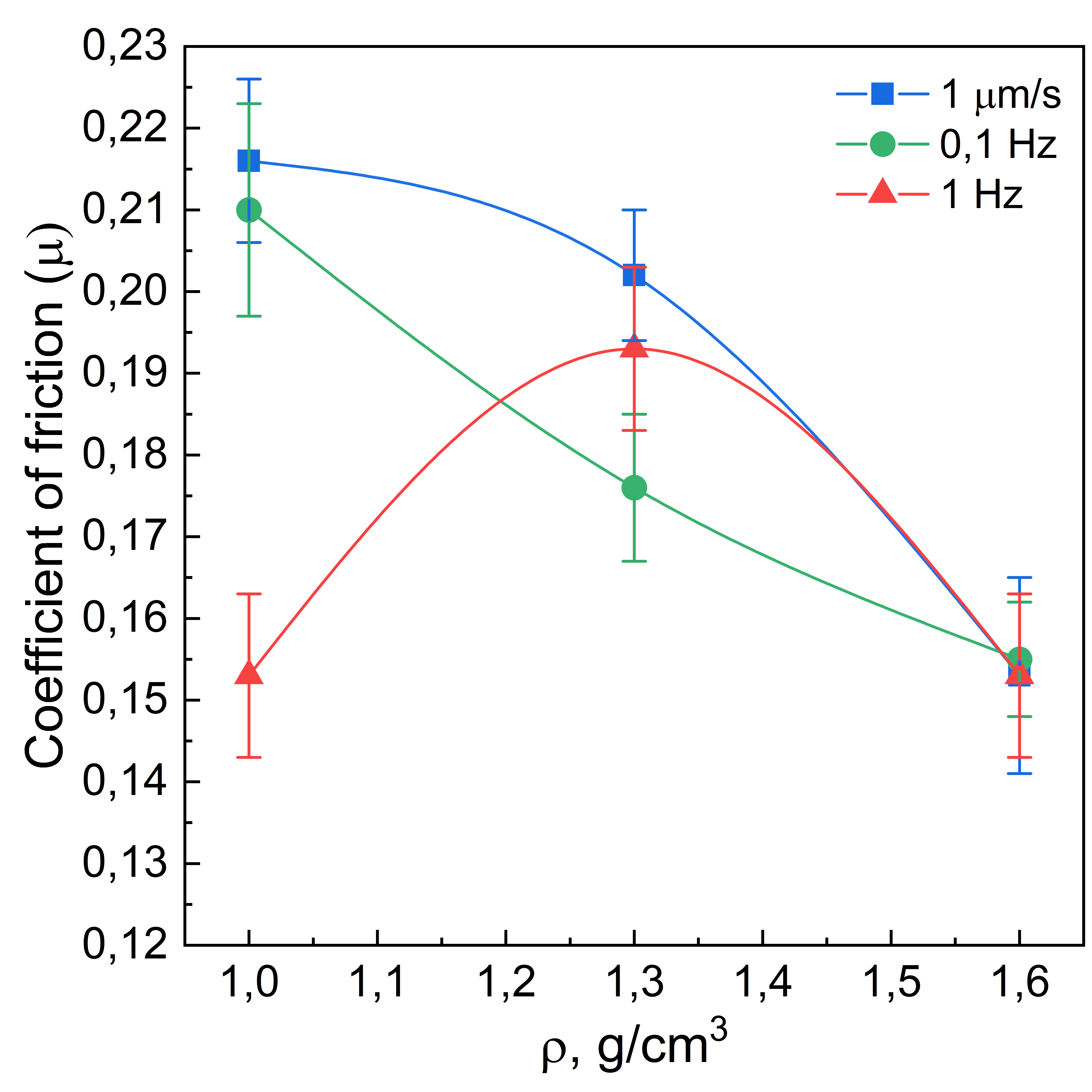

Supplement: Supplementary file 1 [file nanomaterials-14-01499-s001.zip › Tribology/friction coefficient/160-200.png]

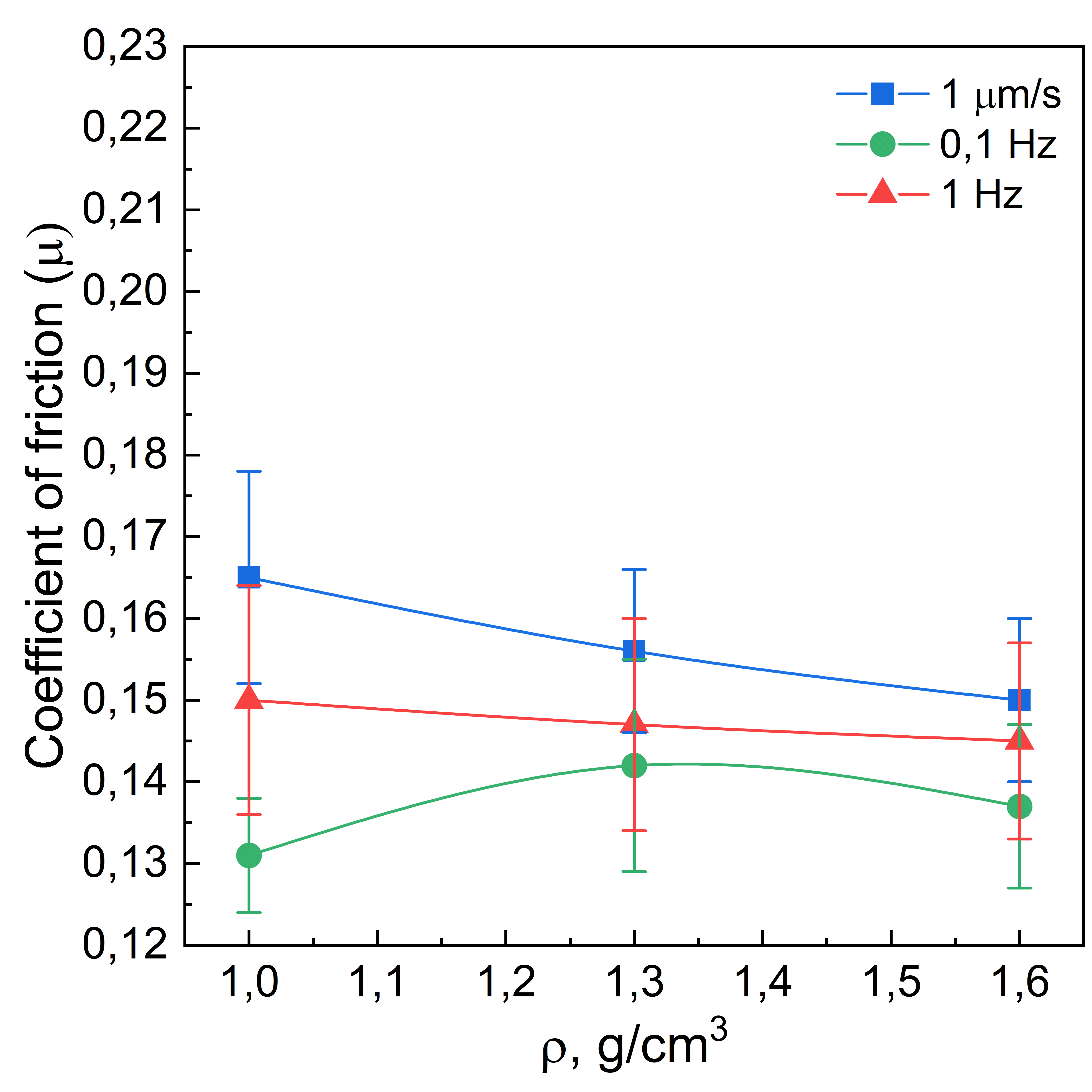

Supplement: Supplementary file 1 [file nanomaterials-14-01499-s001.zip › Tribology/friction coefficient/40-80.png]

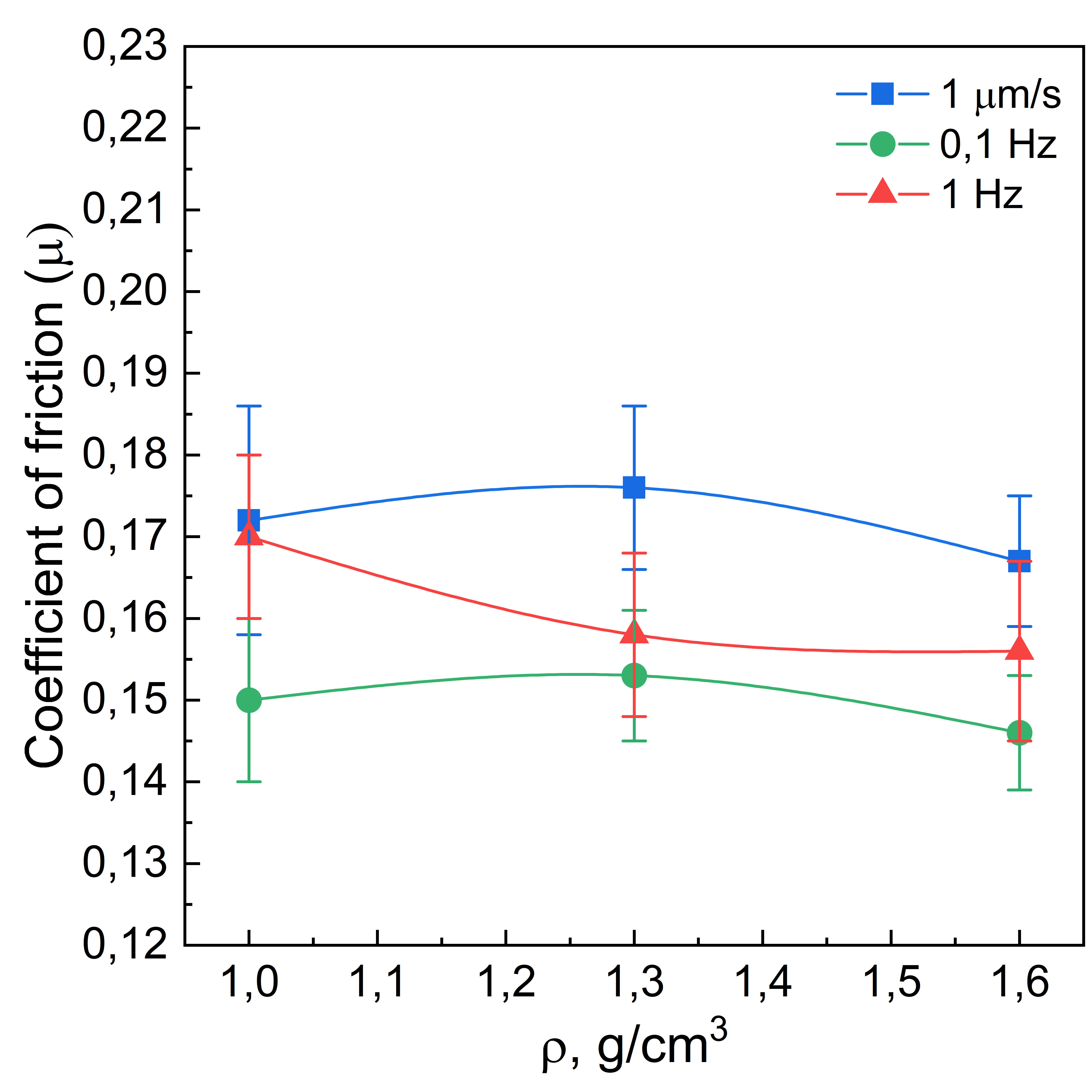

Supplement: Supplementary file 1 [file nanomaterials-14-01499-s001.zip › Tribology/friction coefficient/over500.png]

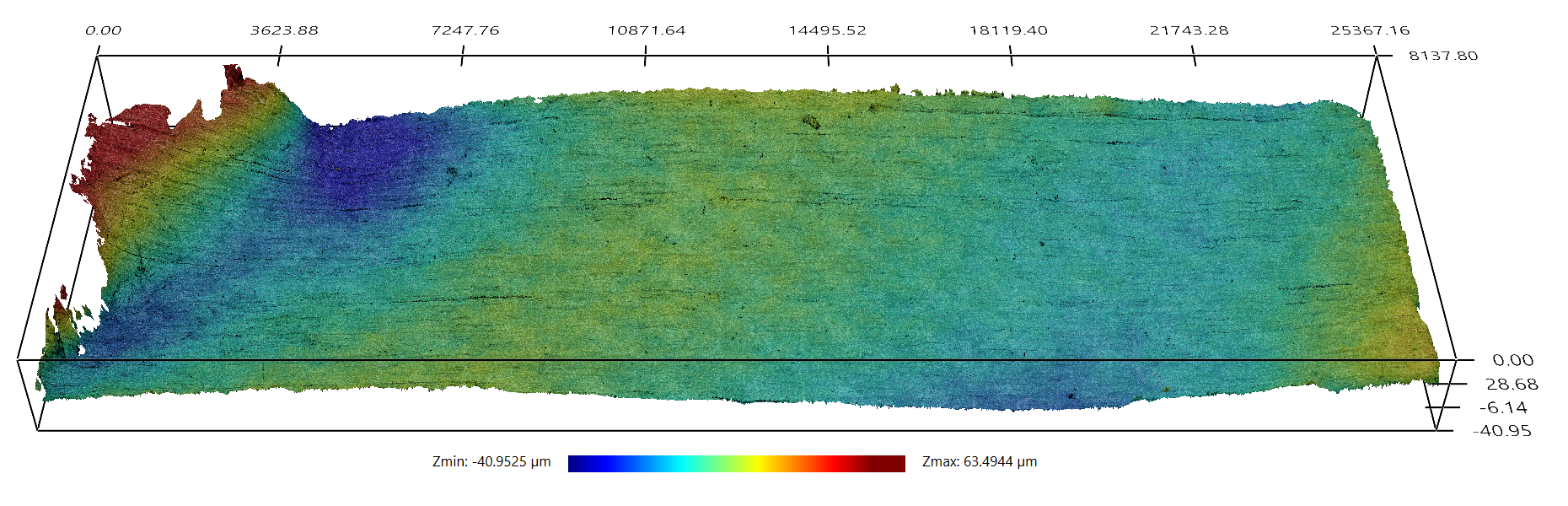

Supplement: Supplementary file 1 [file nanomaterials-14-01499-s001.zip › Tribology/profilograms/0.001/fraction_160-200_density_1_0.png]

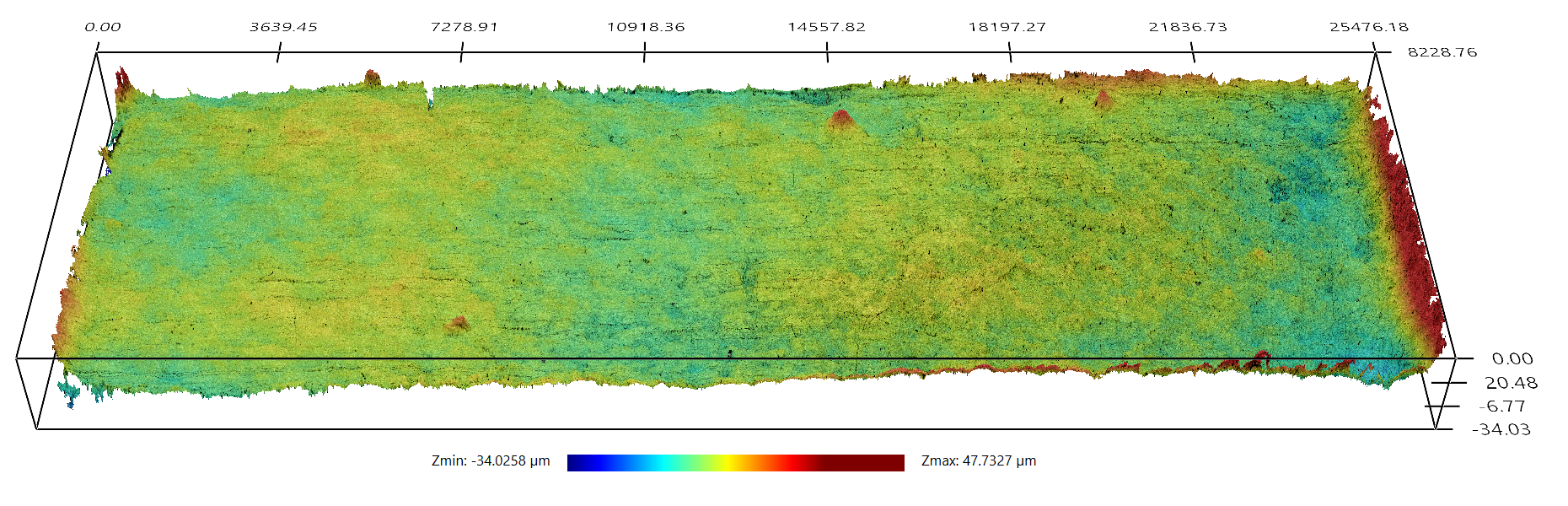

Supplement: Supplementary file 1 [file nanomaterials-14-01499-s001.zip › Tribology/profilograms/0.001/fraction_160-200_density_1_3.png]

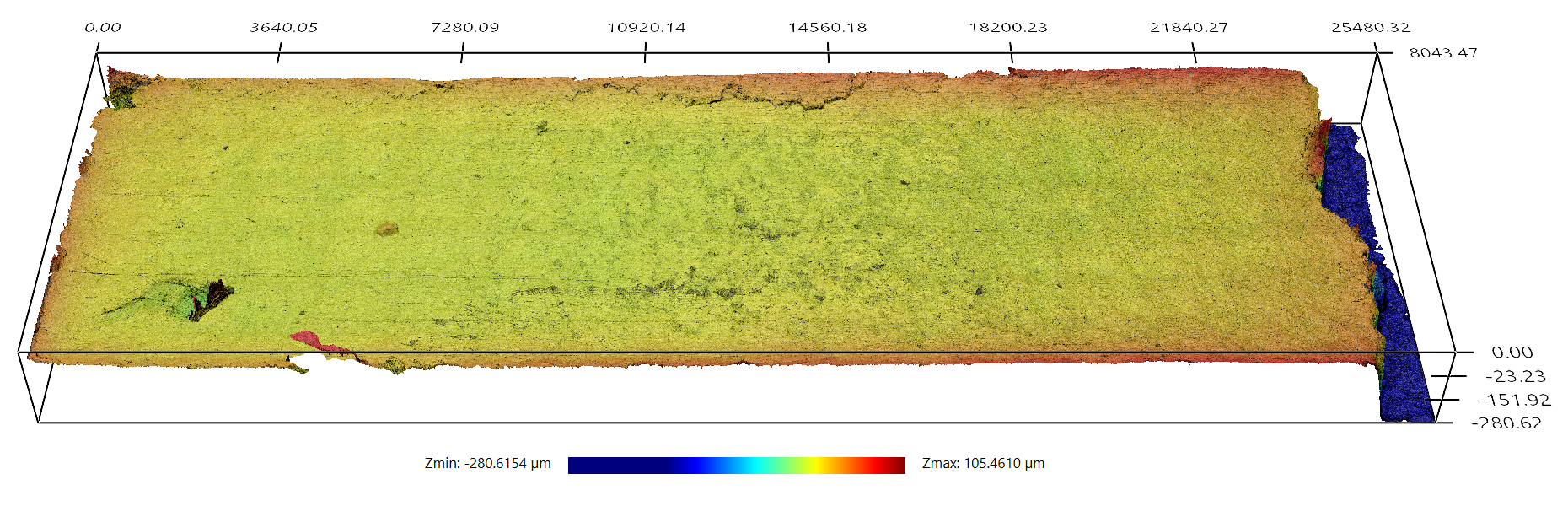

Supplement: Supplementary file 1 [file nanomaterials-14-01499-s001.zip › Tribology/profilograms/0.001/fraction_160-200_density_1_6.png]

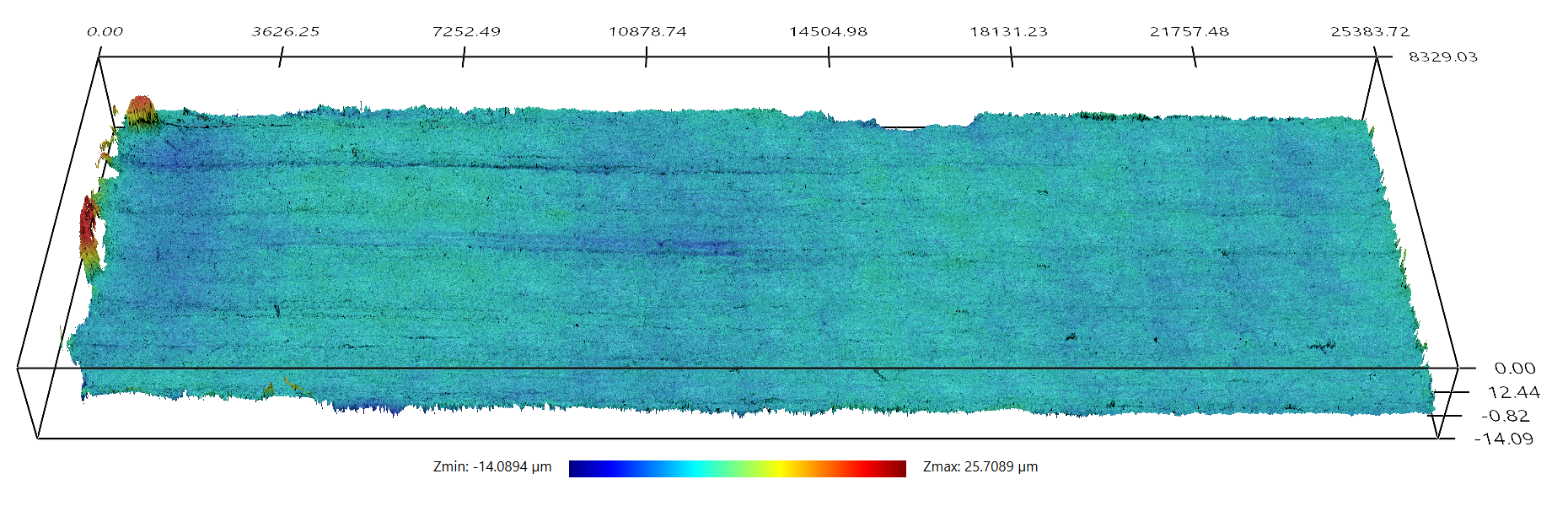

Supplement: Supplementary file 1 [file nanomaterials-14-01499-s001.zip › Tribology/profilograms/0.001/fraction_40-80_density_1_0.png]

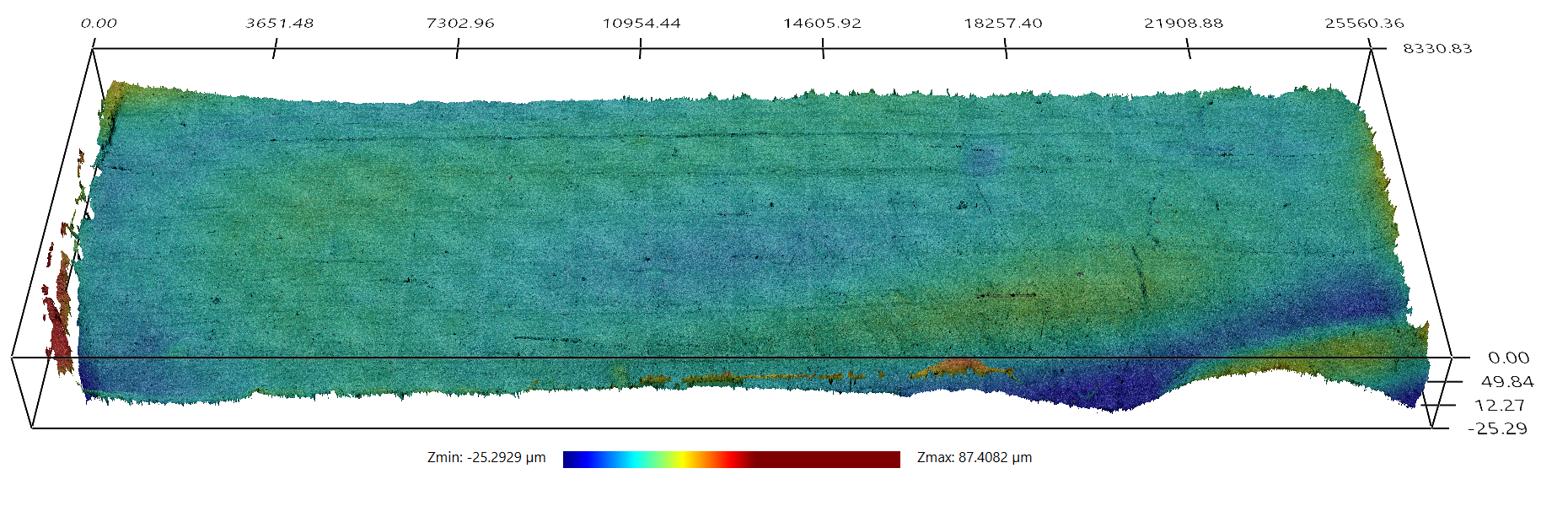

Supplement: Supplementary file 1 [file nanomaterials-14-01499-s001.zip › Tribology/profilograms/0.001/fraction_40-80_density_1_3.png]

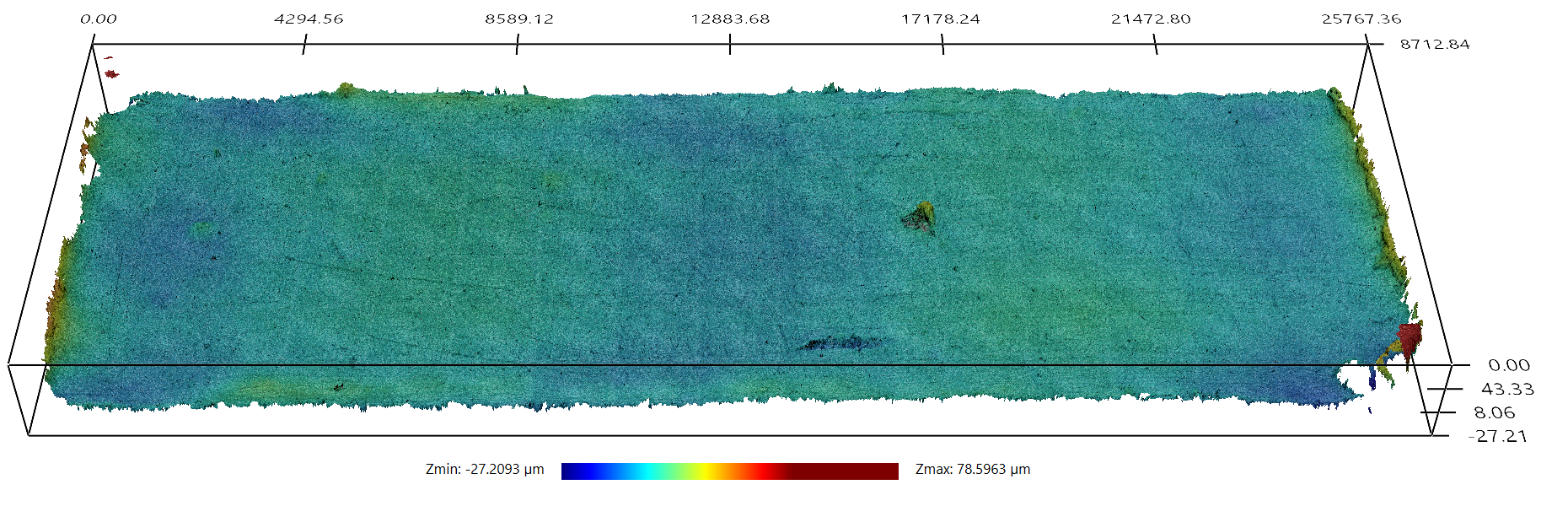

Supplement: Supplementary file 1 [file nanomaterials-14-01499-s001.zip › Tribology/profilograms/0.001/fraction_40-80_density_1_6.png]

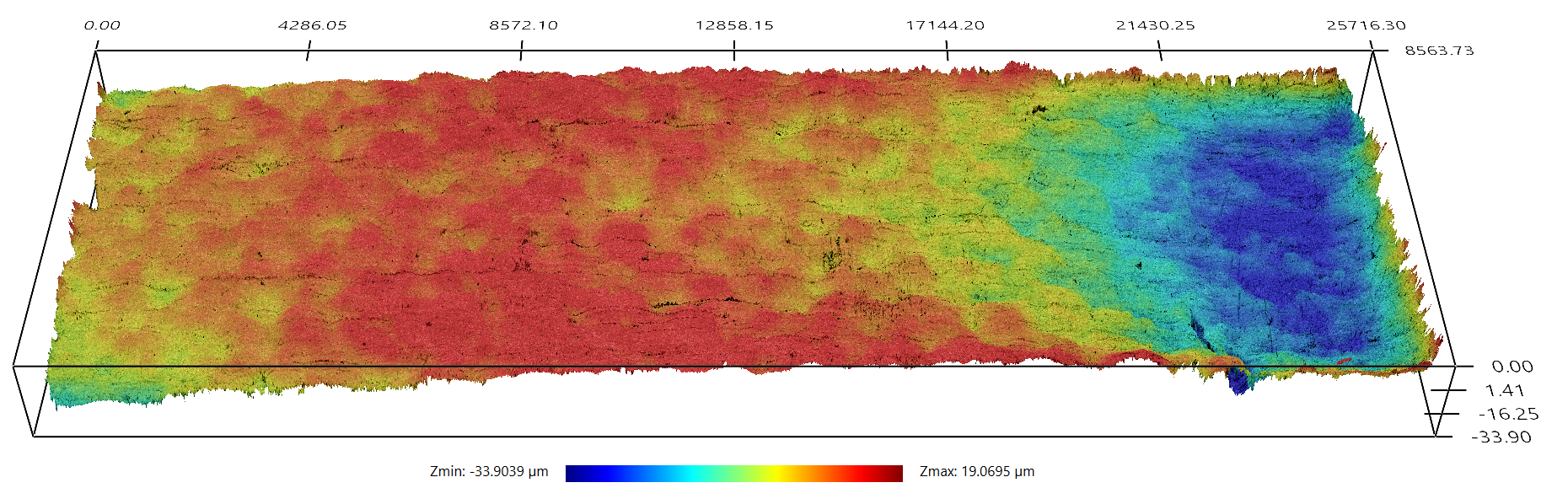

Supplement: Supplementary file 1 [file nanomaterials-14-01499-s001.zip › Tribology/profilograms/0.001/fraction_over500_density1_0.png]

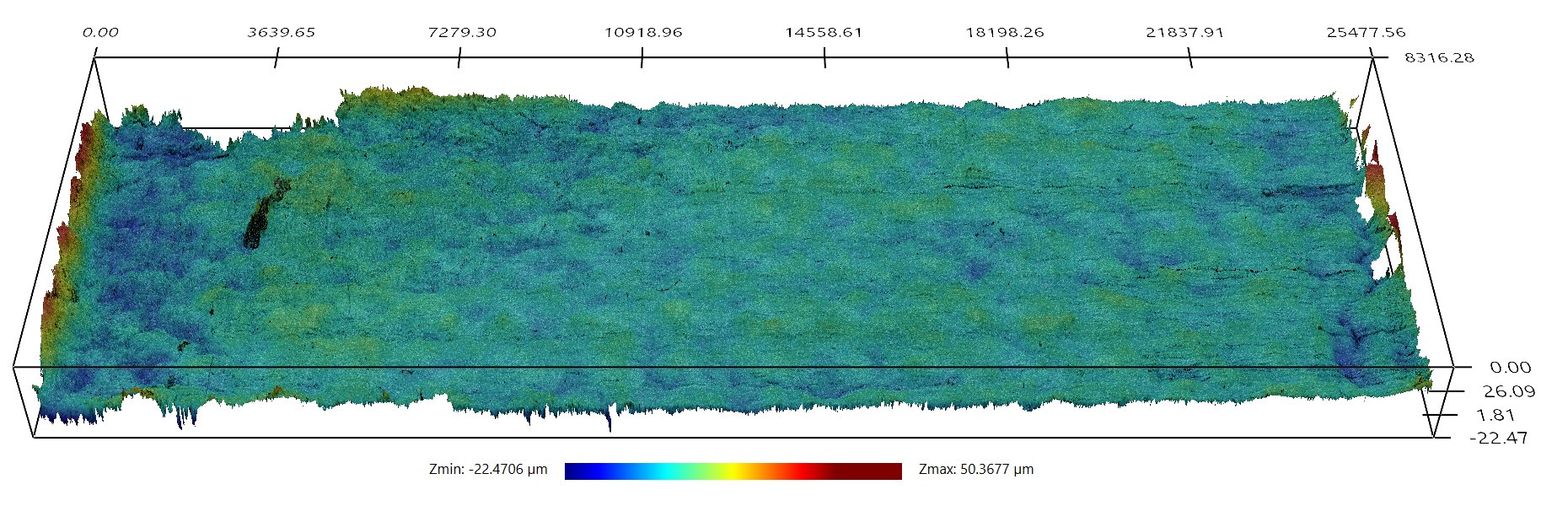

Supplement: Supplementary file 1 [file nanomaterials-14-01499-s001.zip › Tribology/profilograms/0.001/fraction_over500_density1_3.png]

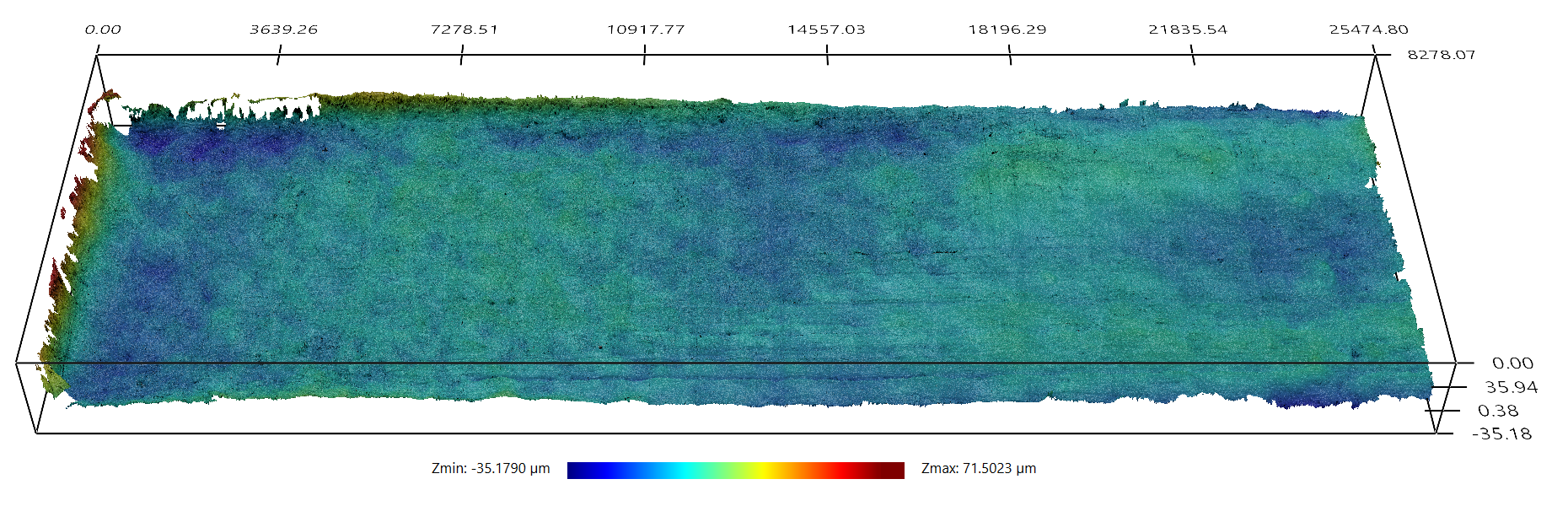

Supplement: Supplementary file 1 [file nanomaterials-14-01499-s001.zip › Tribology/profilograms/0.001/fraction_over500_density1_6.png]

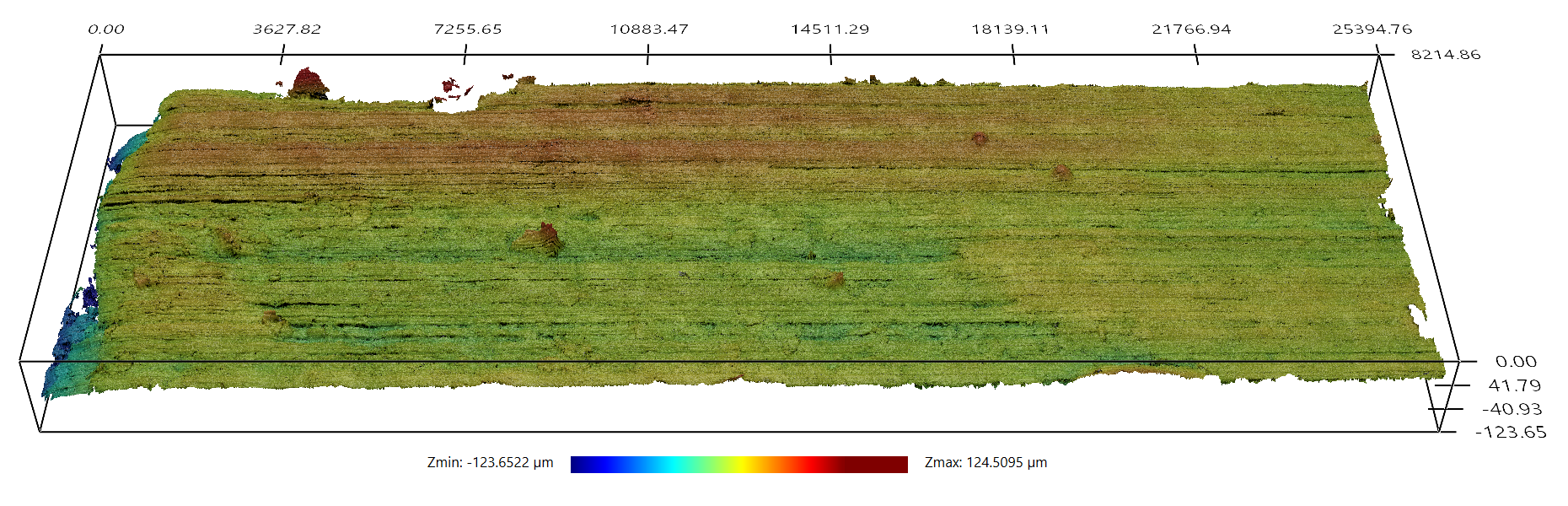

Supplement: Supplementary file 1 [file nanomaterials-14-01499-s001.zip › Tribology/profilograms/0.1Hz/fraction_160-200_density_1_0.png]

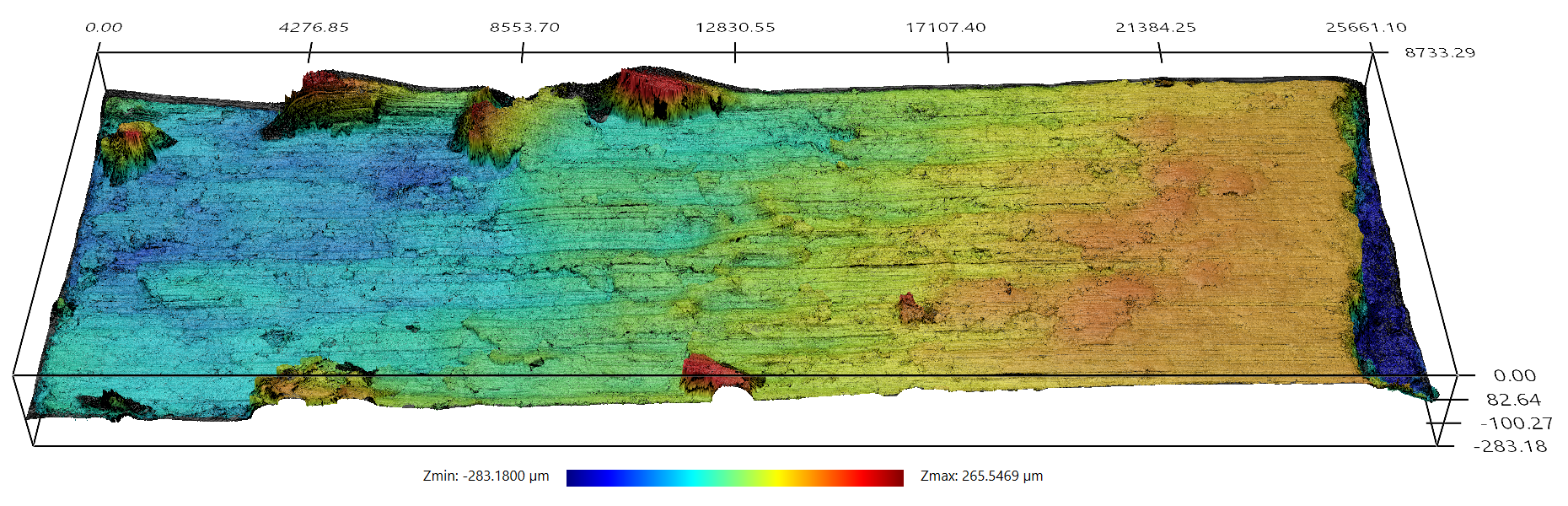

Supplement: Supplementary file 1 [file nanomaterials-14-01499-s001.zip › Tribology/profilograms/0.1Hz/fraction_160-200_density_1_3.png]

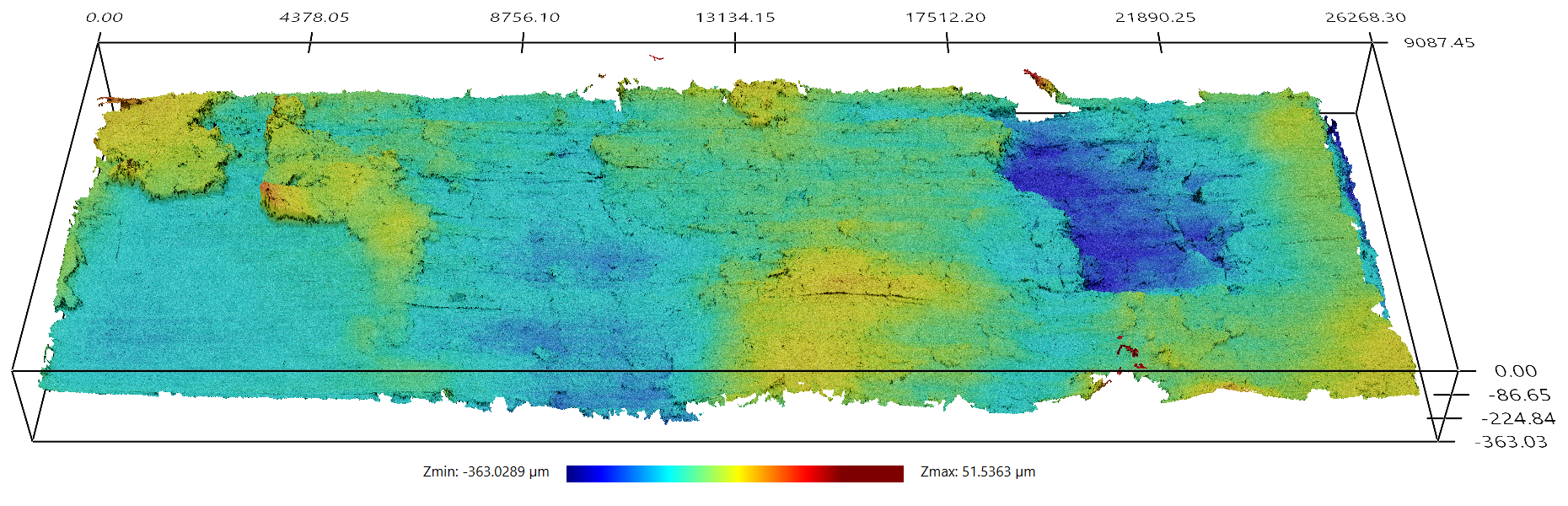

Supplement: Supplementary file 1 [file nanomaterials-14-01499-s001.zip › Tribology/profilograms/0.1Hz/fraction_160-200_density_1_6.png]

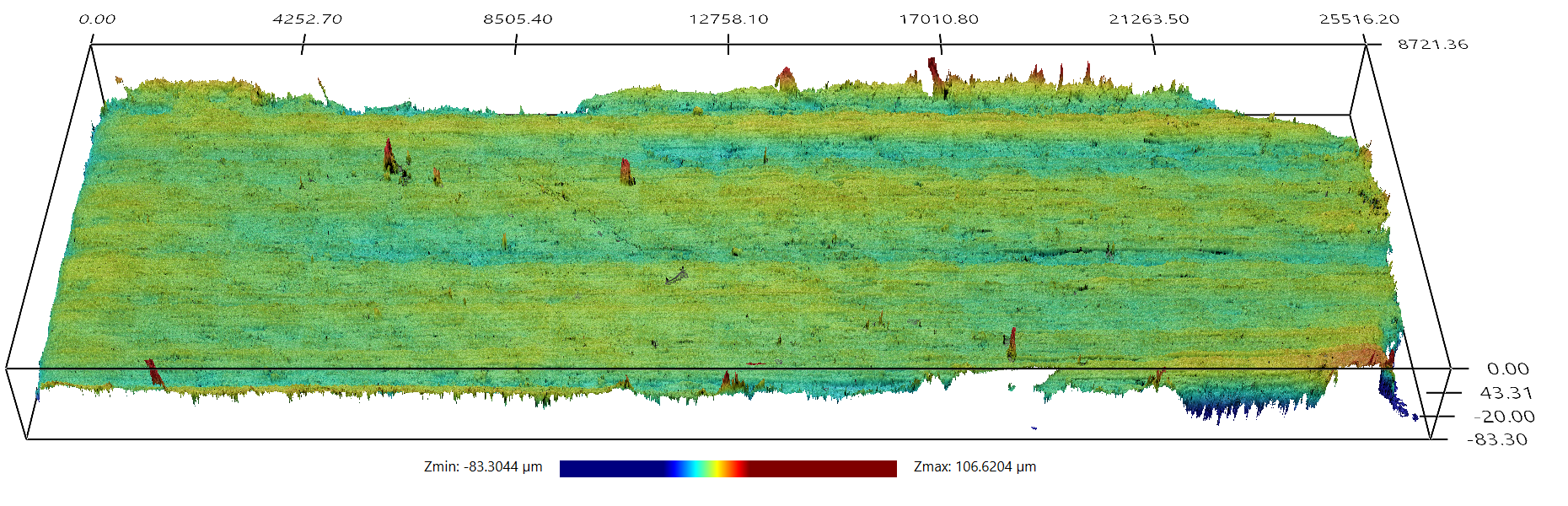

Supplement: Supplementary file 1 [file nanomaterials-14-01499-s001.zip › Tribology/profilograms/0.1Hz/fraction_40-80_density_1_0.png]

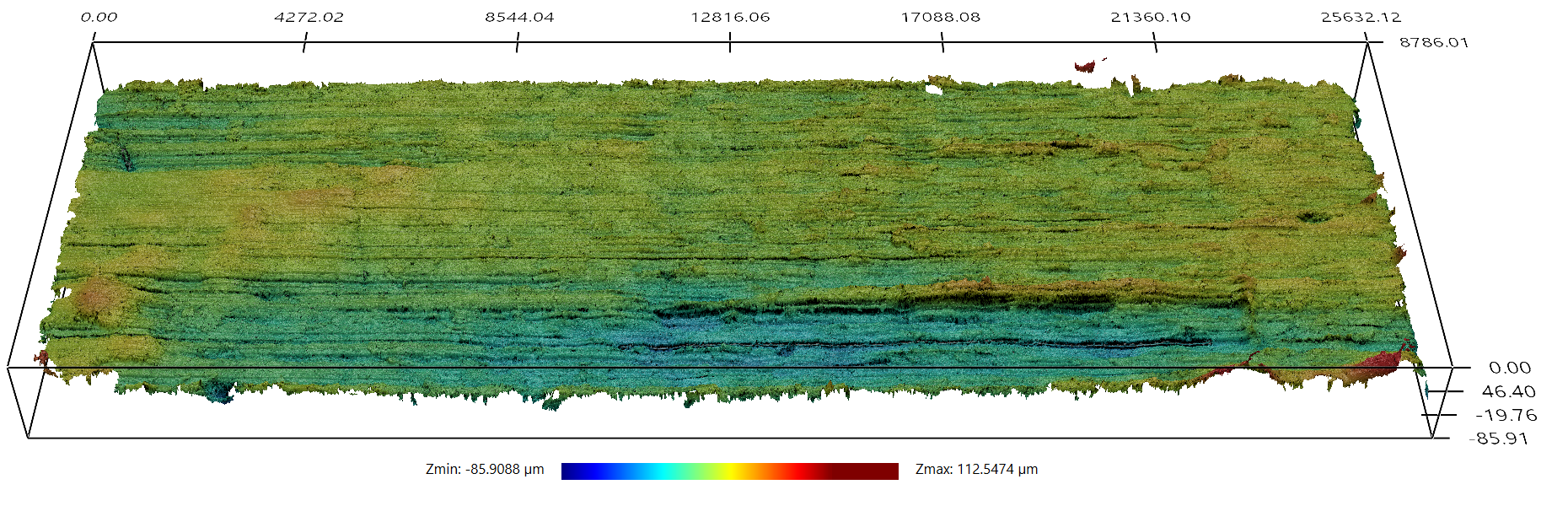

Supplement: Supplementary file 1 [file nanomaterials-14-01499-s001.zip › Tribology/profilograms/0.1Hz/fraction_40-80_density_1_3.png]

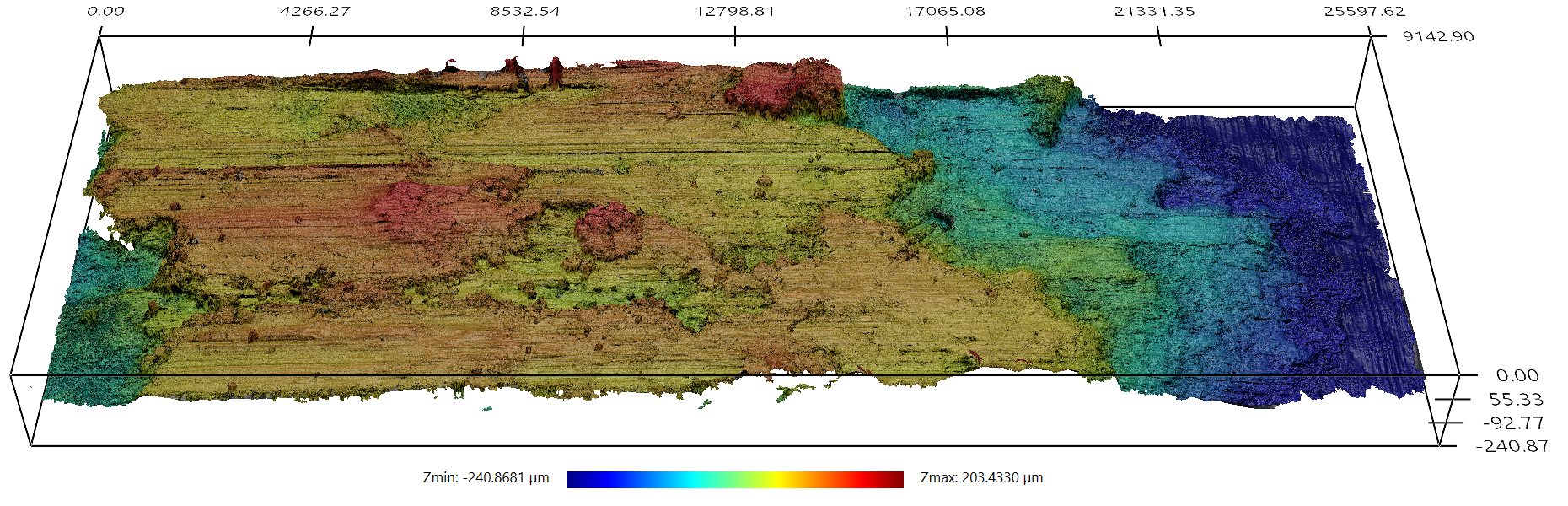

Supplement: Supplementary file 1 [file nanomaterials-14-01499-s001.zip › Tribology/profilograms/0.1Hz/fraction_40-80_density_1_6.png]

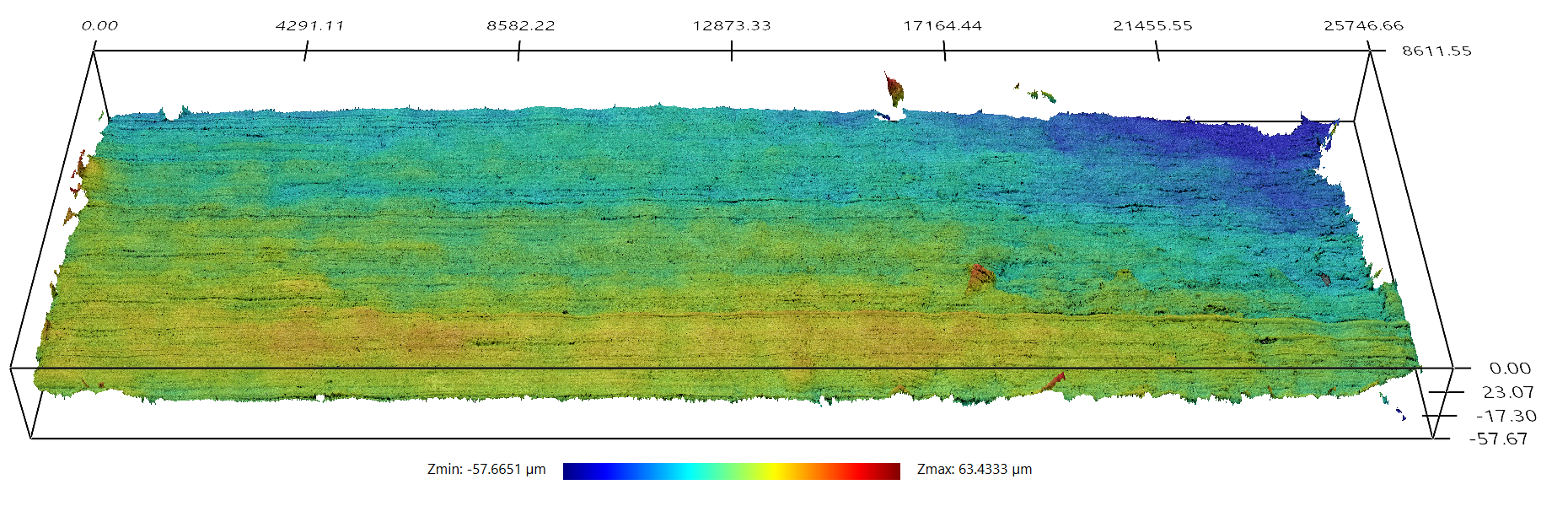

Supplement: Supplementary file 1 [file nanomaterials-14-01499-s001.zip › Tribology/profilograms/0.1Hz/fraction_over500_density1_0.png]

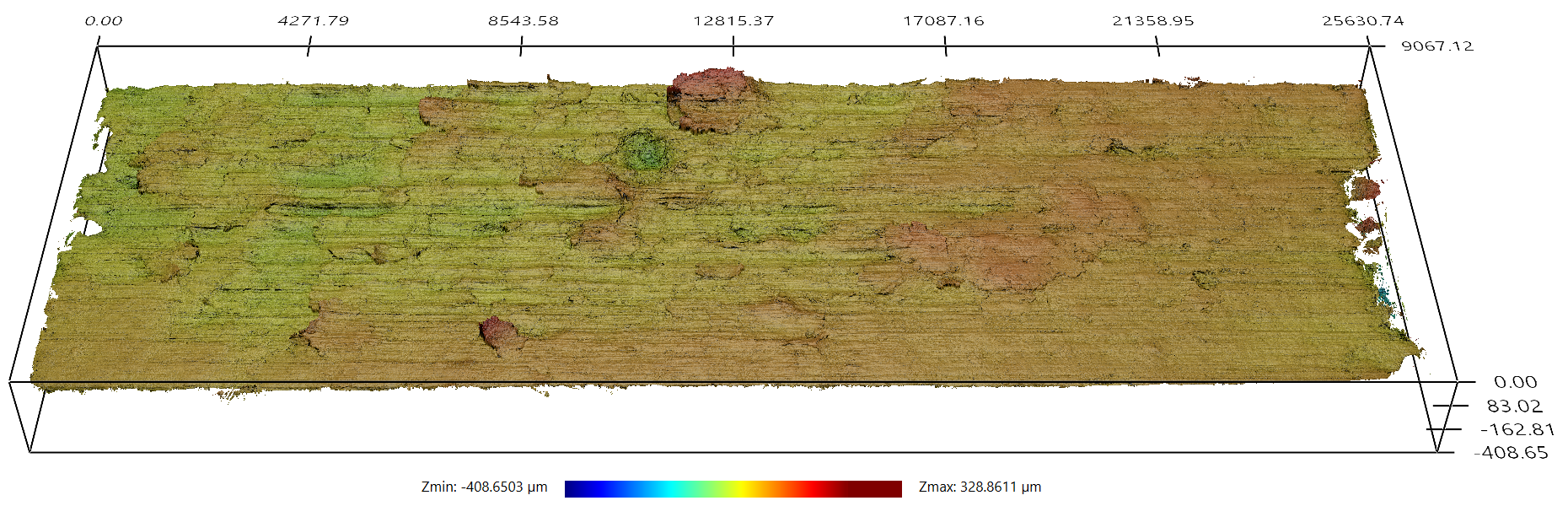

Supplement: Supplementary file 1 [file nanomaterials-14-01499-s001.zip › Tribology/profilograms/0.1Hz/fraction_over500_density1_3.png]

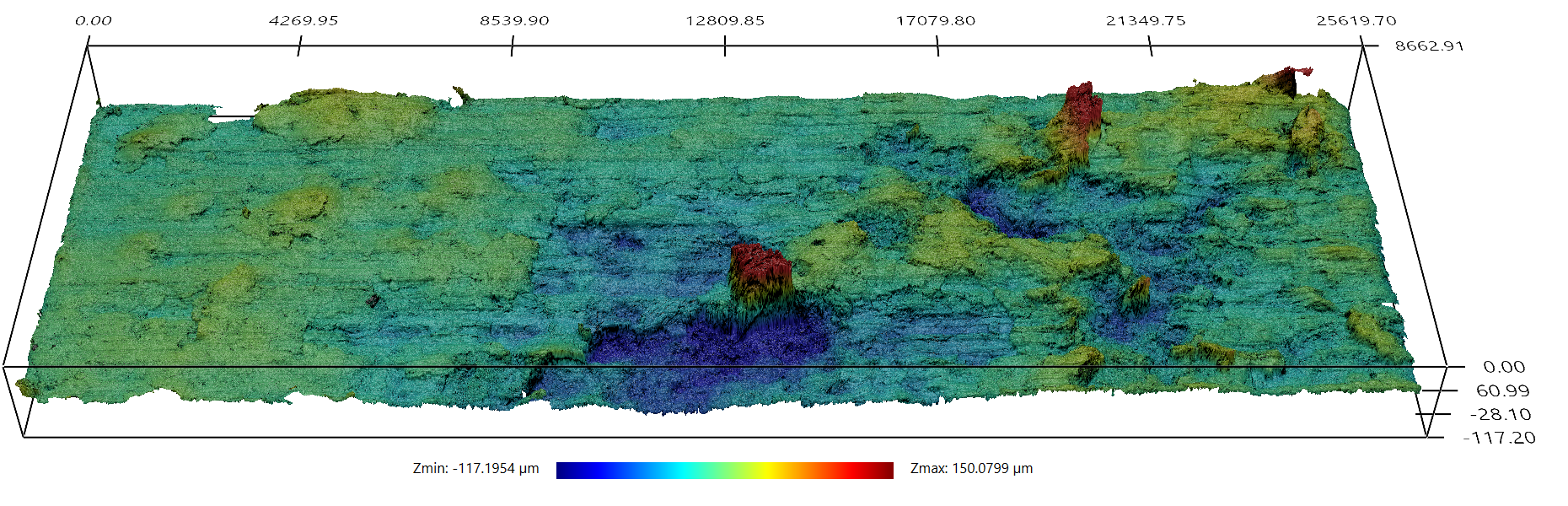

Supplement: Supplementary file 1 [file nanomaterials-14-01499-s001.zip › Tribology/profilograms/0.1Hz/fraction_over500_density1_6.png]

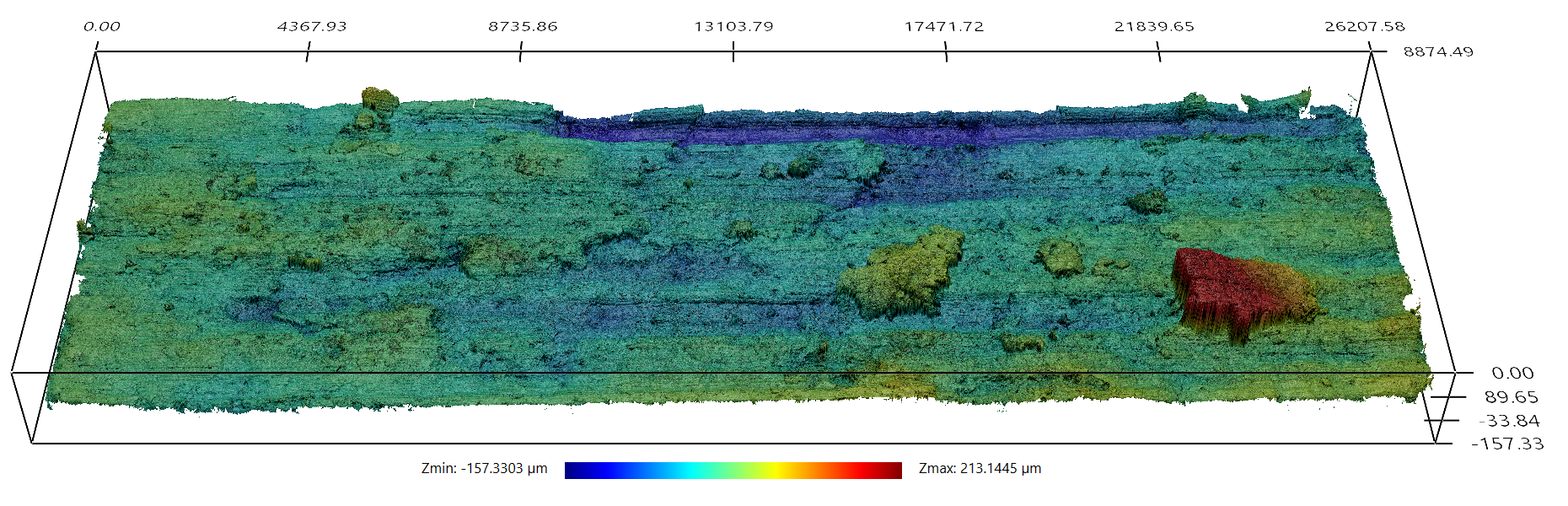

Supplement: Supplementary file 1 [file nanomaterials-14-01499-s001.zip › Tribology/profilograms/1Hz/fraction_160-200_density_1_0.png]

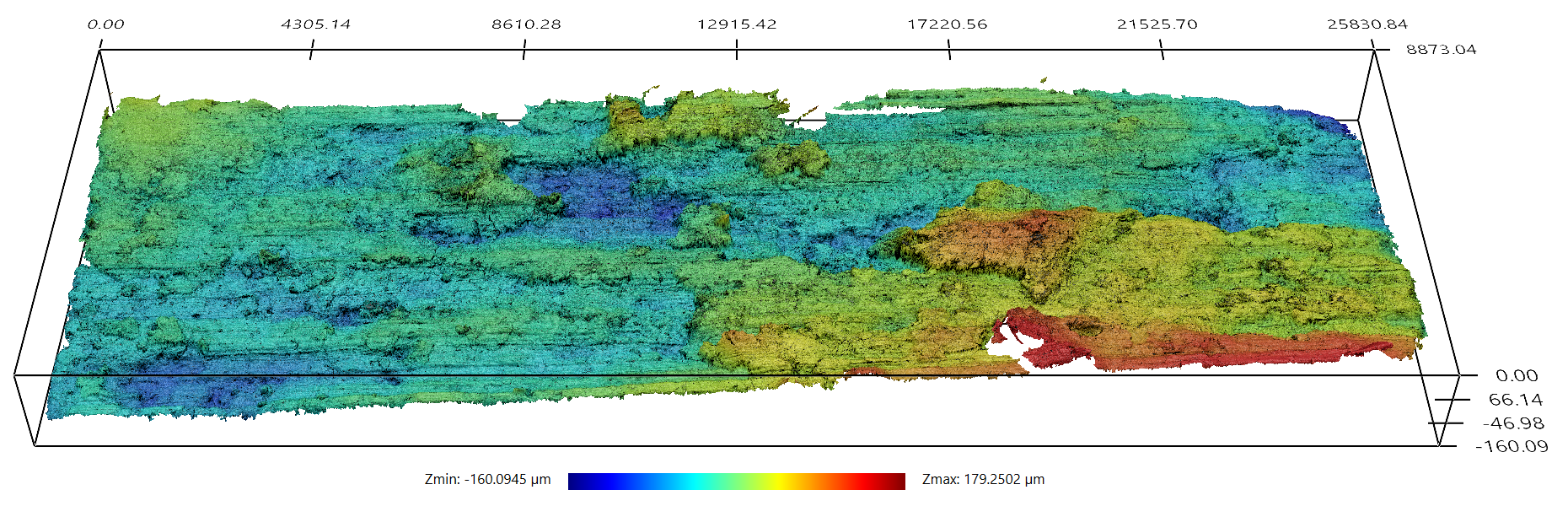

Supplement: Supplementary file 1 [file nanomaterials-14-01499-s001.zip › Tribology/profilograms/1Hz/fraction_160-200_density_1_3.png]

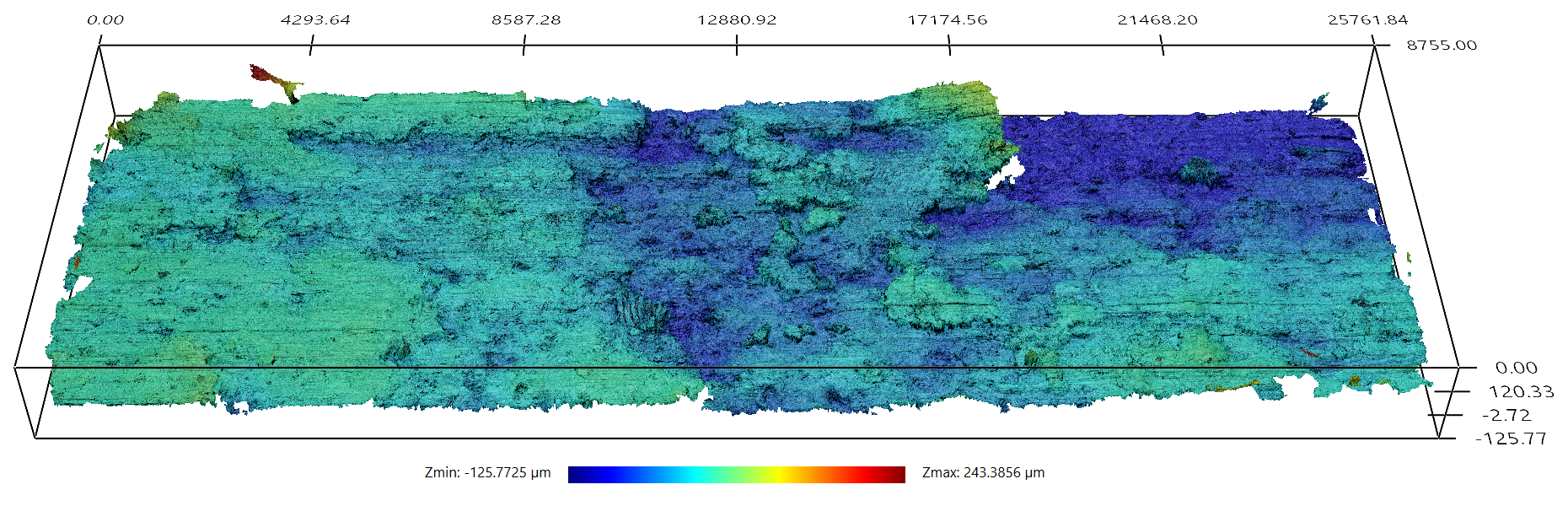

Supplement: Supplementary file 1 [file nanomaterials-14-01499-s001.zip › Tribology/profilograms/1Hz/fraction_160-200_density_1_6.png]

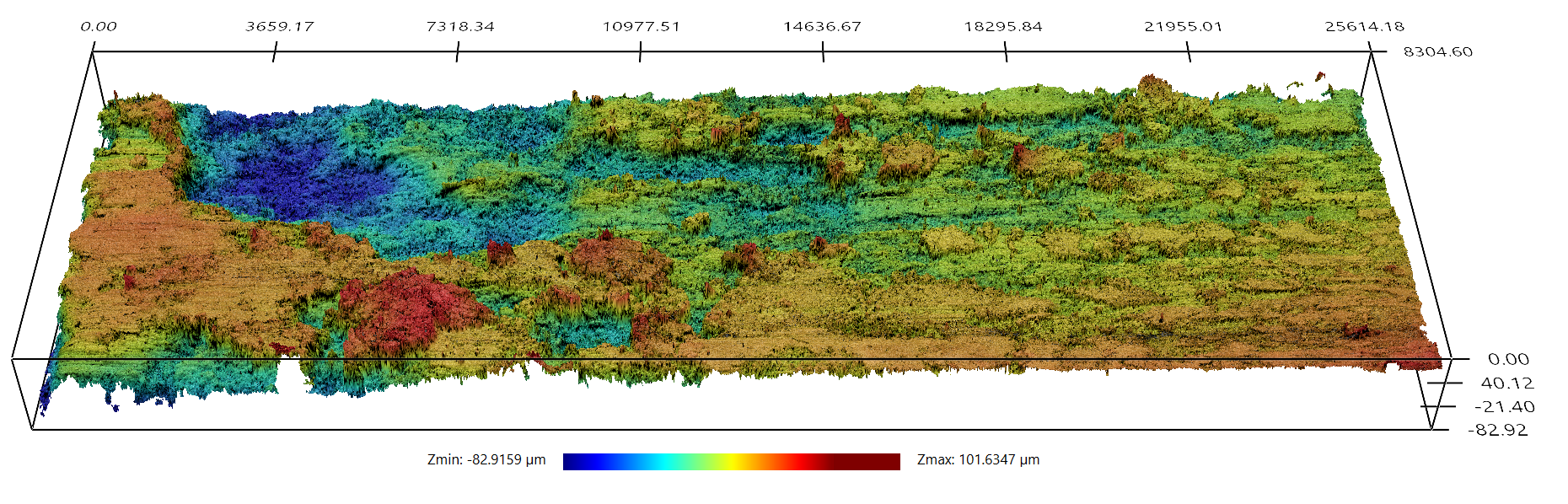

Supplement: Supplementary file 1 [file nanomaterials-14-01499-s001.zip › Tribology/profilograms/1Hz/fraction_40-80_density_1_0.png]

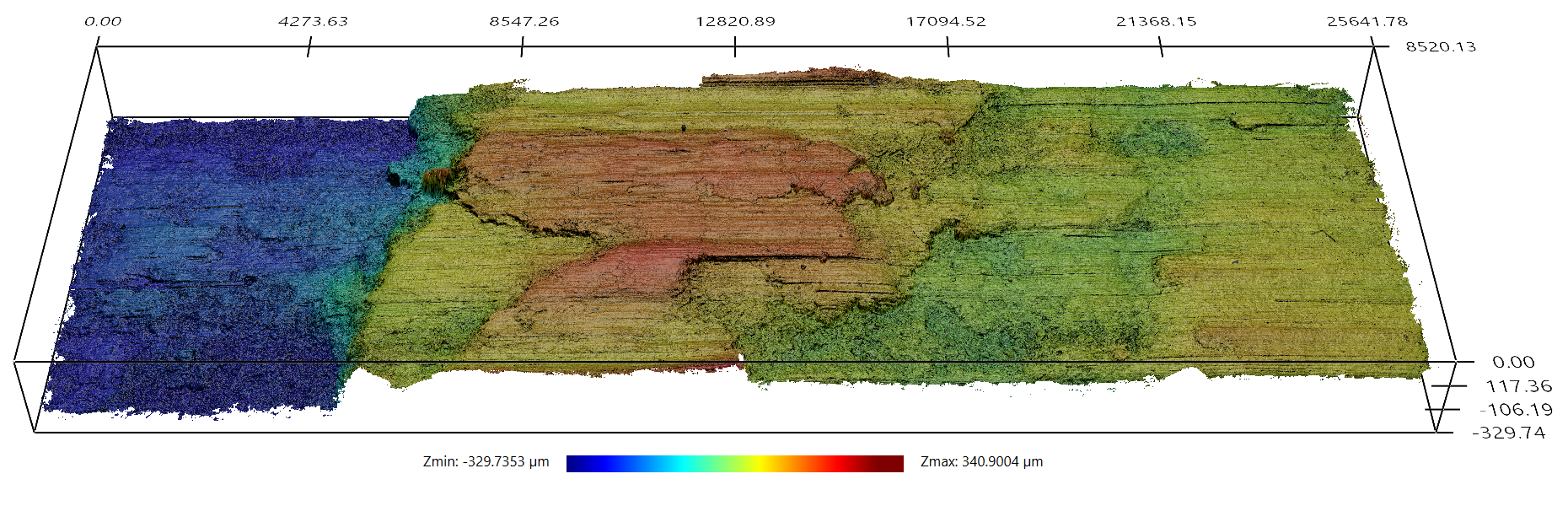

Supplement: Supplementary file 1 [file nanomaterials-14-01499-s001.zip › Tribology/profilograms/1Hz/fraction_40-80_density_1_3.png]

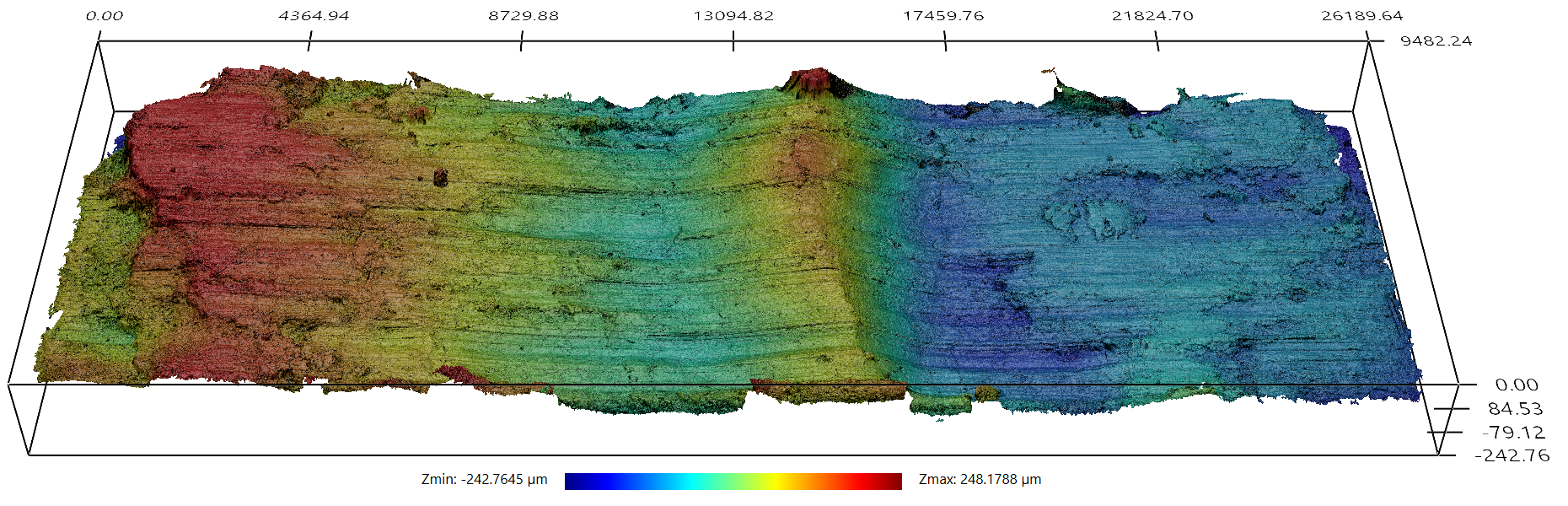

Supplement: Supplementary file 1 [file nanomaterials-14-01499-s001.zip › Tribology/profilograms/1Hz/fraction_40-80_density_1_6.png]

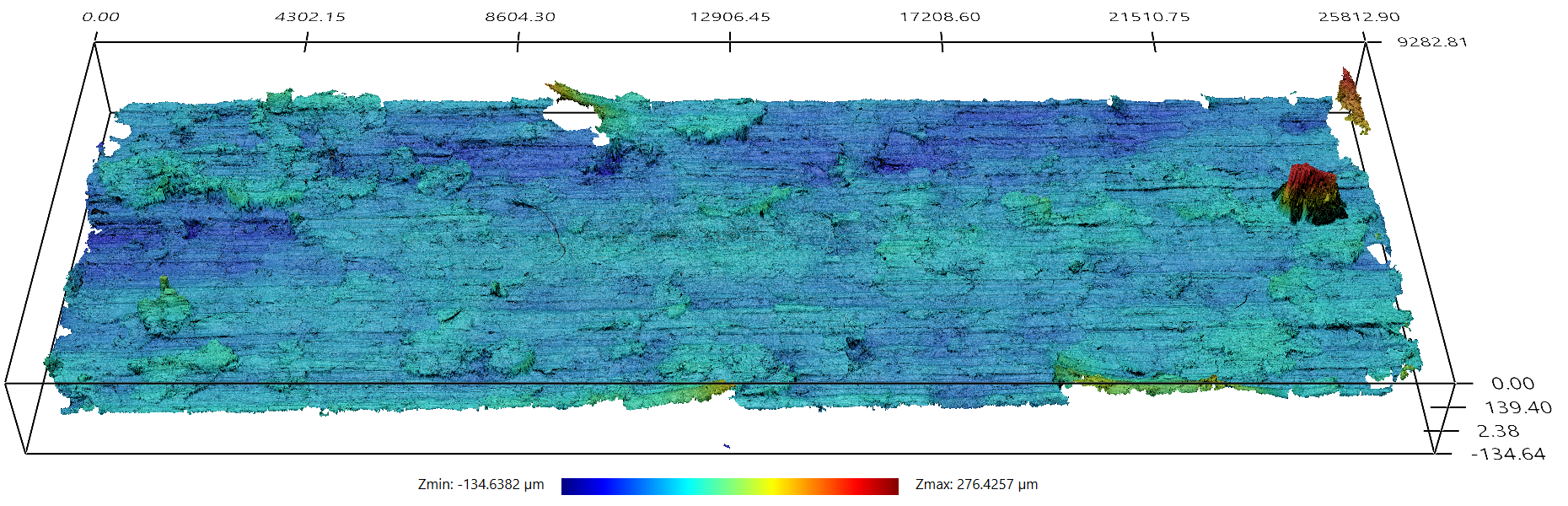

Supplement: Supplementary file 1 [file nanomaterials-14-01499-s001.zip › Tribology/profilograms/1Hz/fraction_over500_density1_0.png]

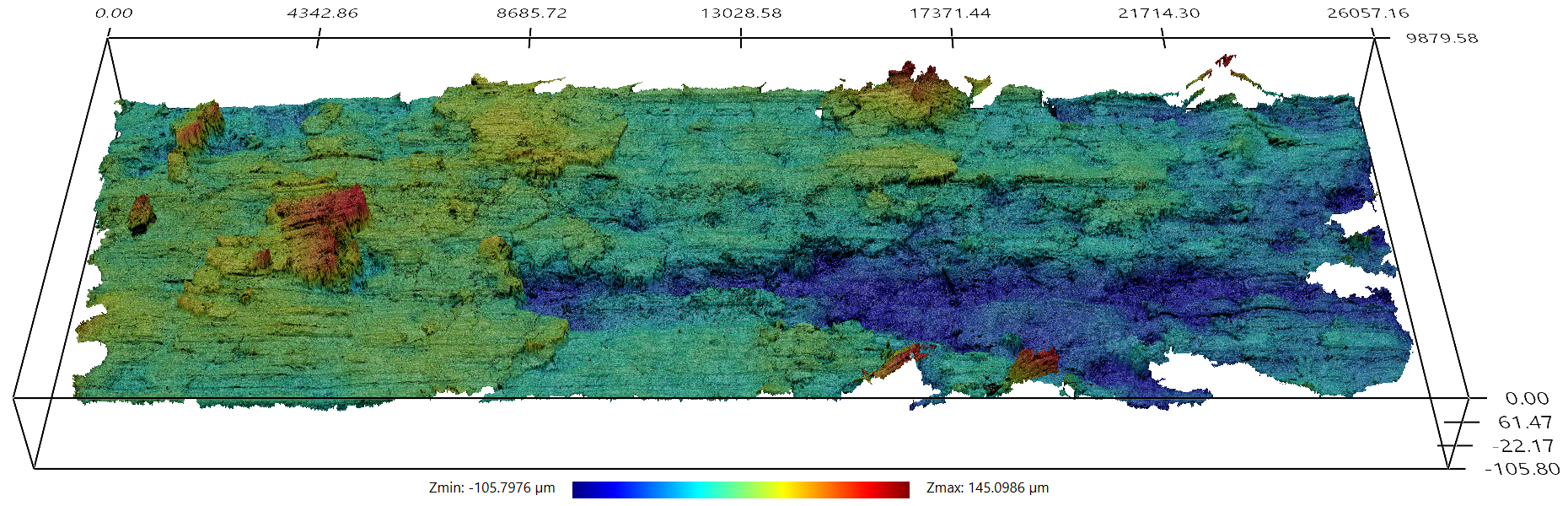

Supplement: Supplementary file 1 [file nanomaterials-14-01499-s001.zip › Tribology/profilograms/1Hz/fraction_over500_density1_3.png]

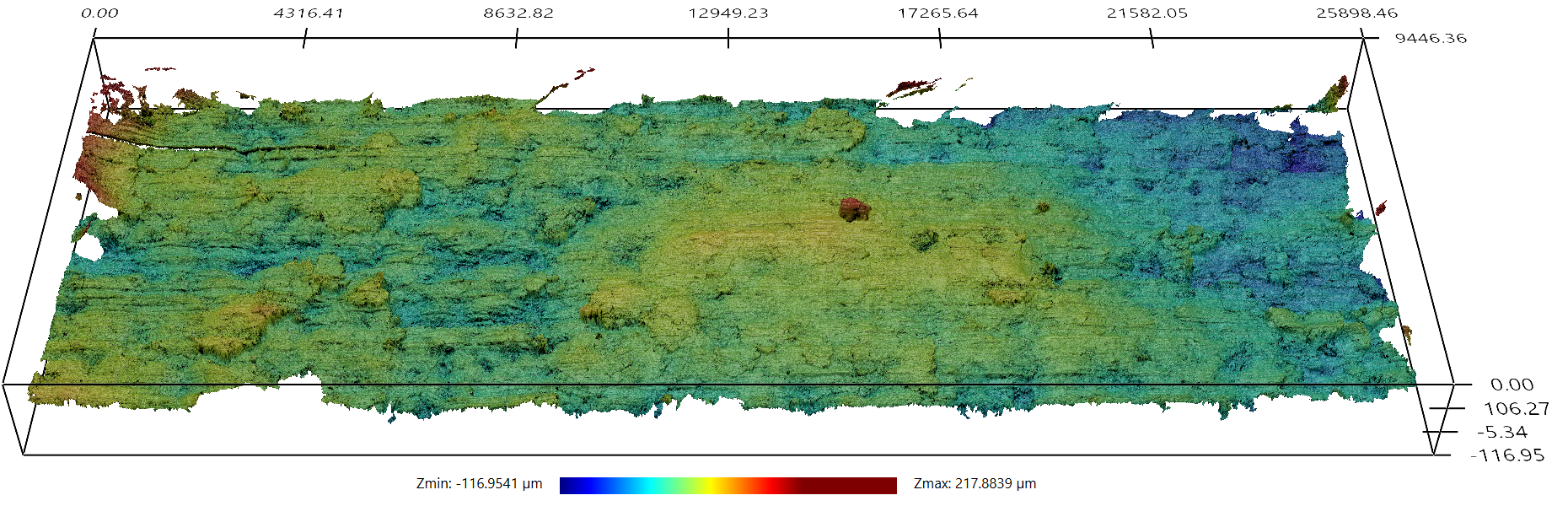

Supplement: Supplementary file 1 [file nanomaterials-14-01499-s001.zip › Tribology/profilograms/1Hz/fraction_over500_density1_6.png]

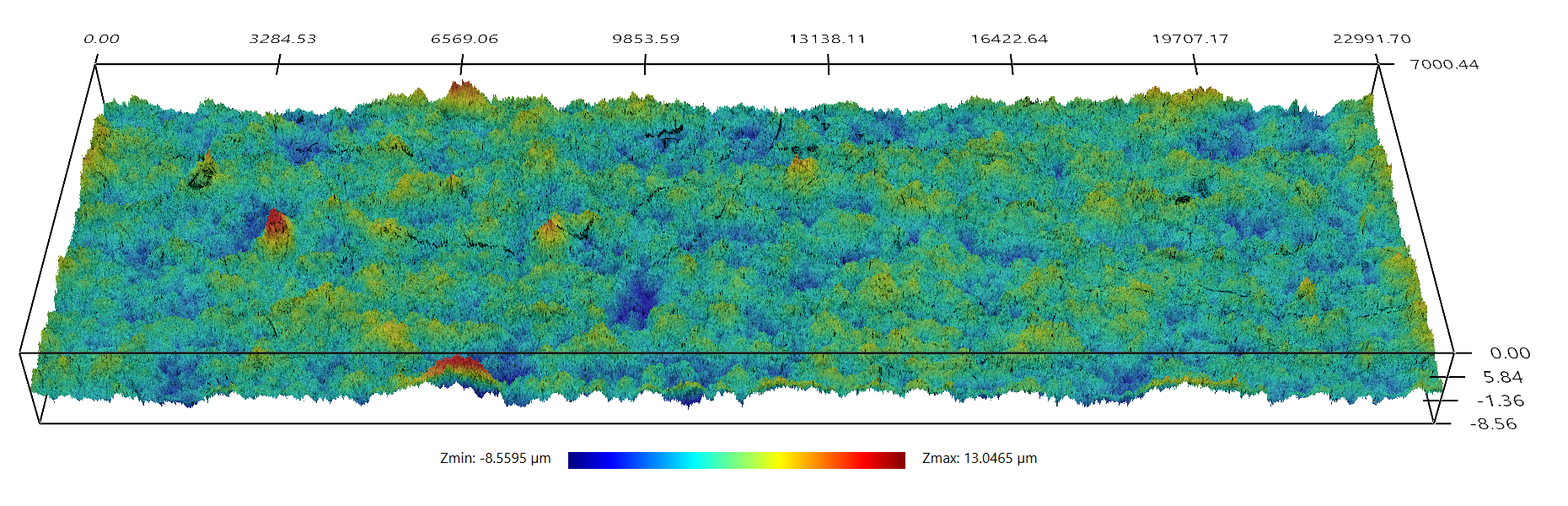

Supplement: Supplementary file 1 [file nanomaterials-14-01499-s001.zip › Tribology/profilograms/basic/fraction_160-200_density_1_0.png]

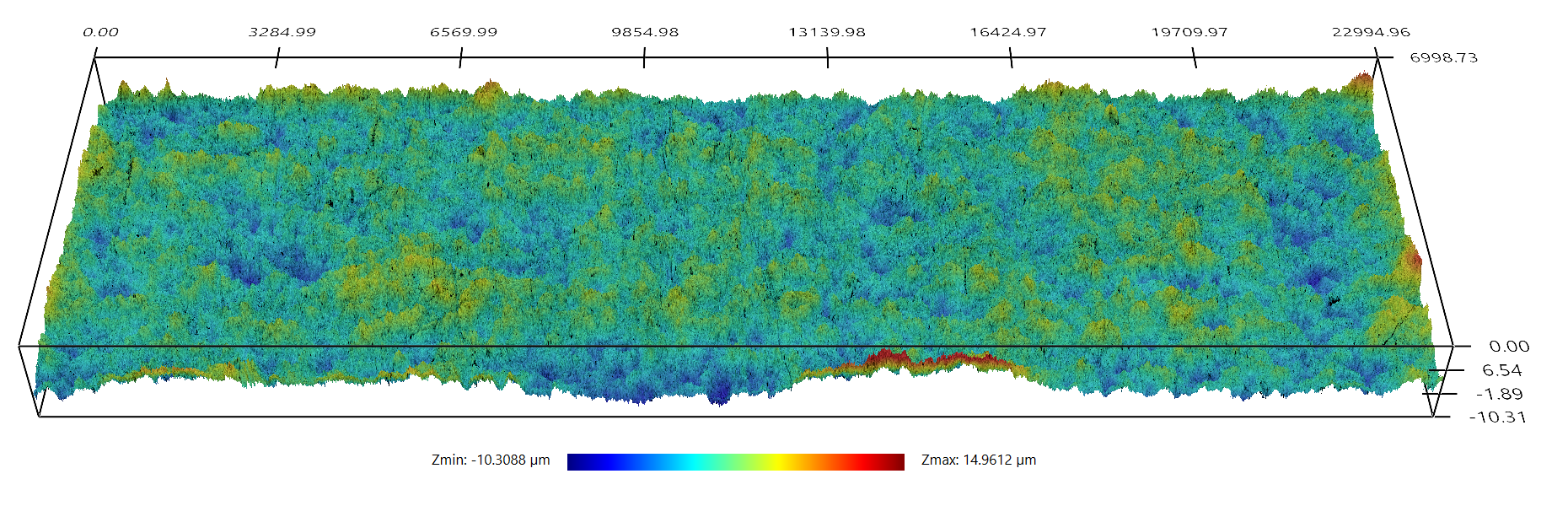

Supplement: Supplementary file 1 [file nanomaterials-14-01499-s001.zip › Tribology/profilograms/basic/fraction_160-200_density_1_3.png]

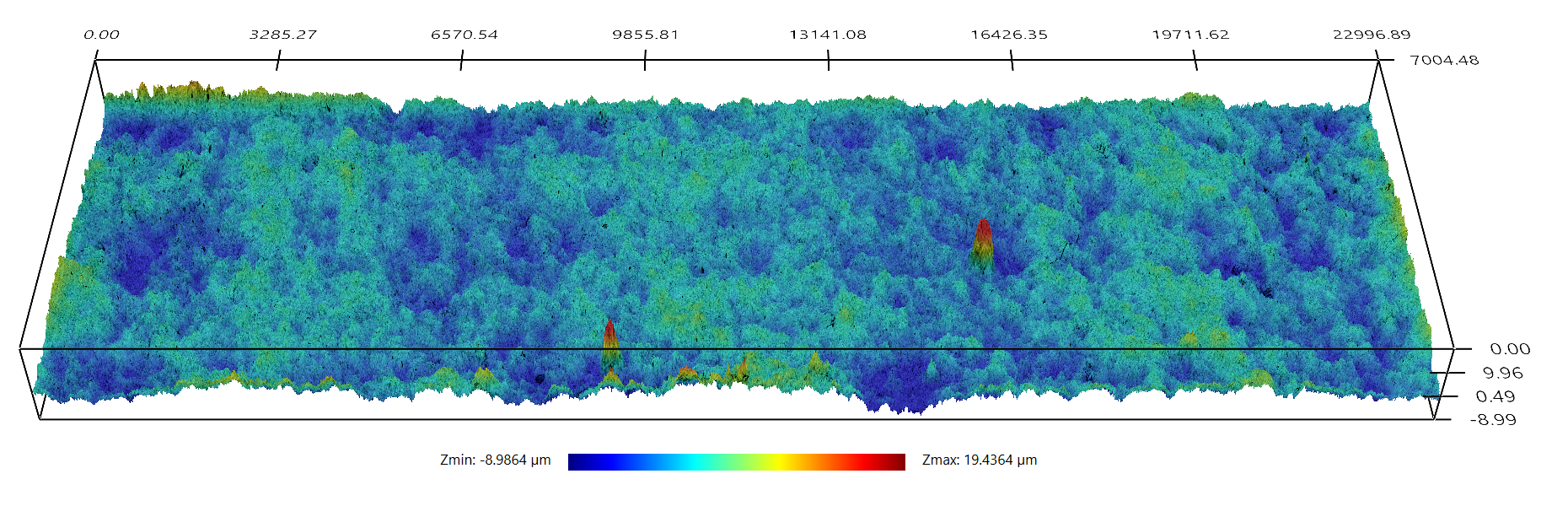

Supplement: Supplementary file 1 [file nanomaterials-14-01499-s001.zip › Tribology/profilograms/basic/fraction_160-200_density_1_6.png]

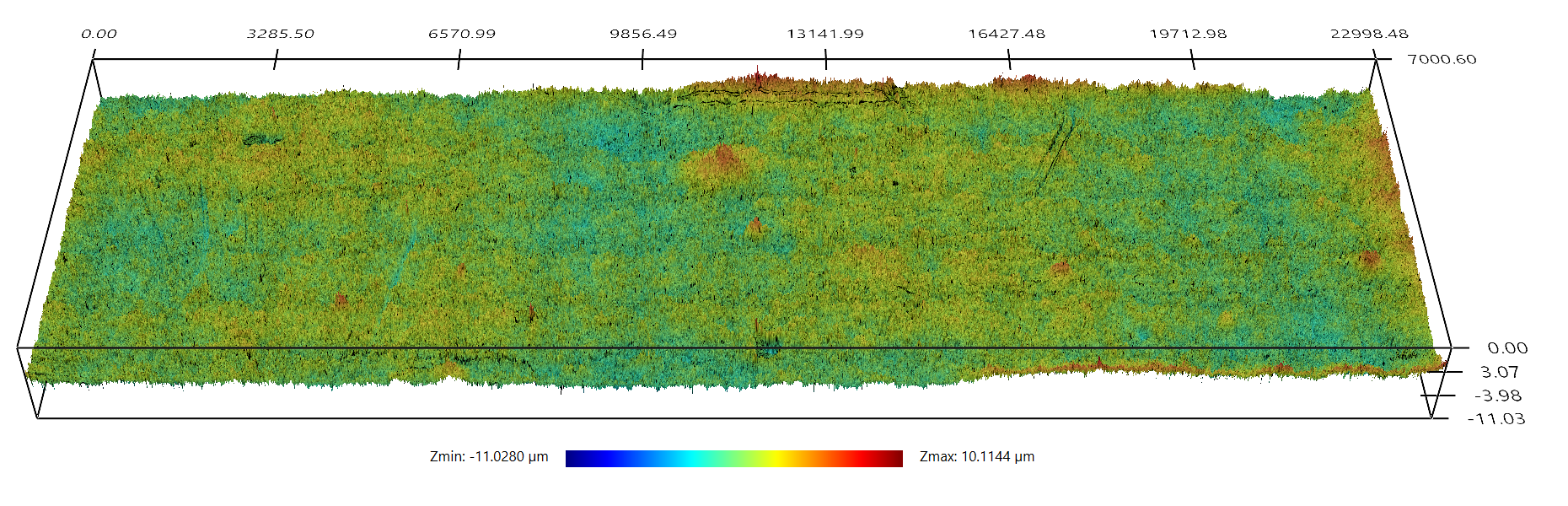

Supplement: Supplementary file 1 [file nanomaterials-14-01499-s001.zip › Tribology/profilograms/basic/fraction_40-80_density_1_0.png]

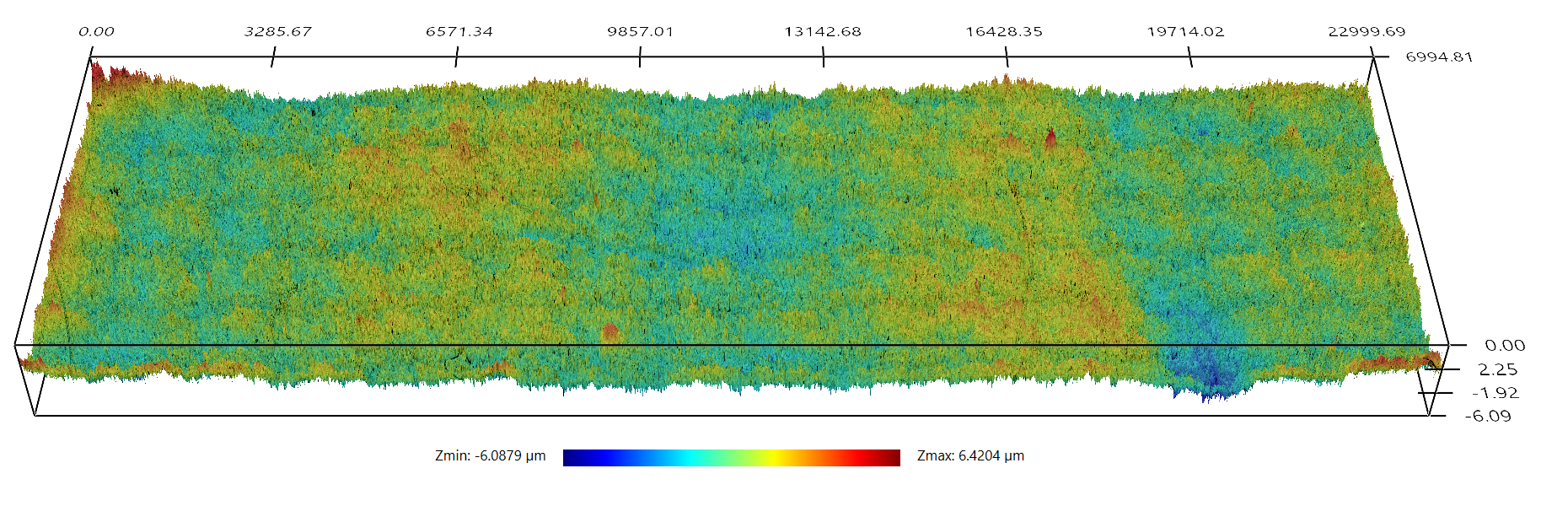

Supplement: Supplementary file 1 [file nanomaterials-14-01499-s001.zip › Tribology/profilograms/basic/fraction_40-80_density_1_3.png]

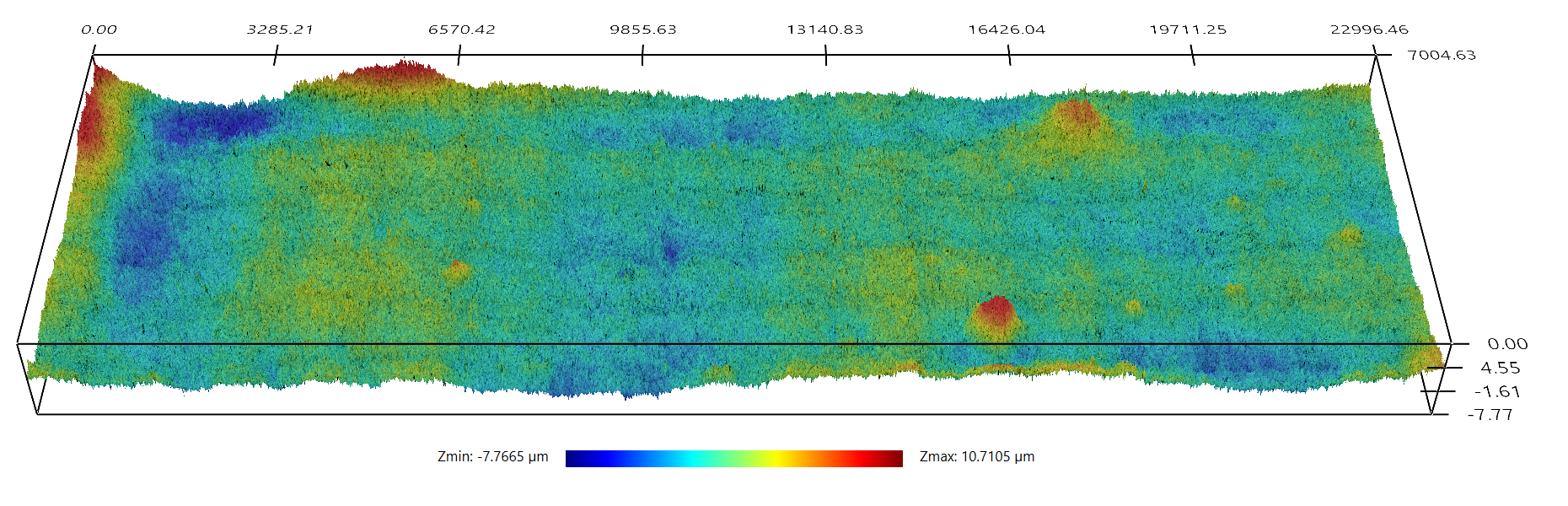

Supplement: Supplementary file 1 [file nanomaterials-14-01499-s001.zip › Tribology/profilograms/basic/fraction_40-80_density_1_6.png]

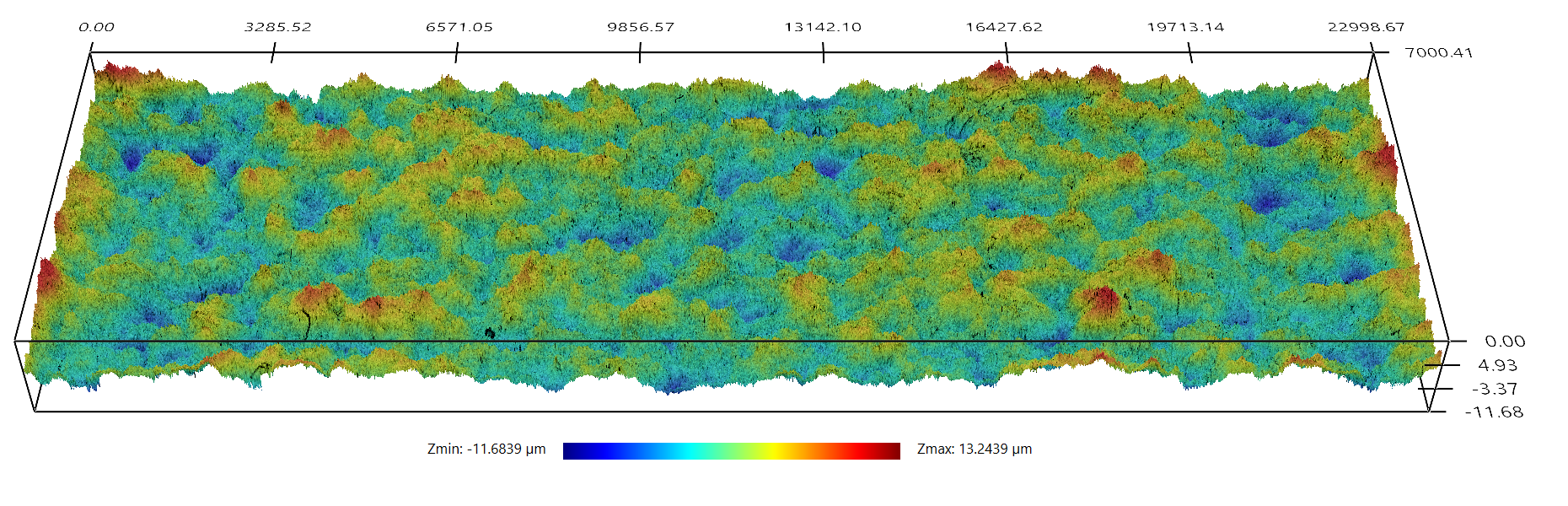

Supplement: Supplementary file 1 [file nanomaterials-14-01499-s001.zip › Tribology/profilograms/basic/fraction_over500_density1_0.png]

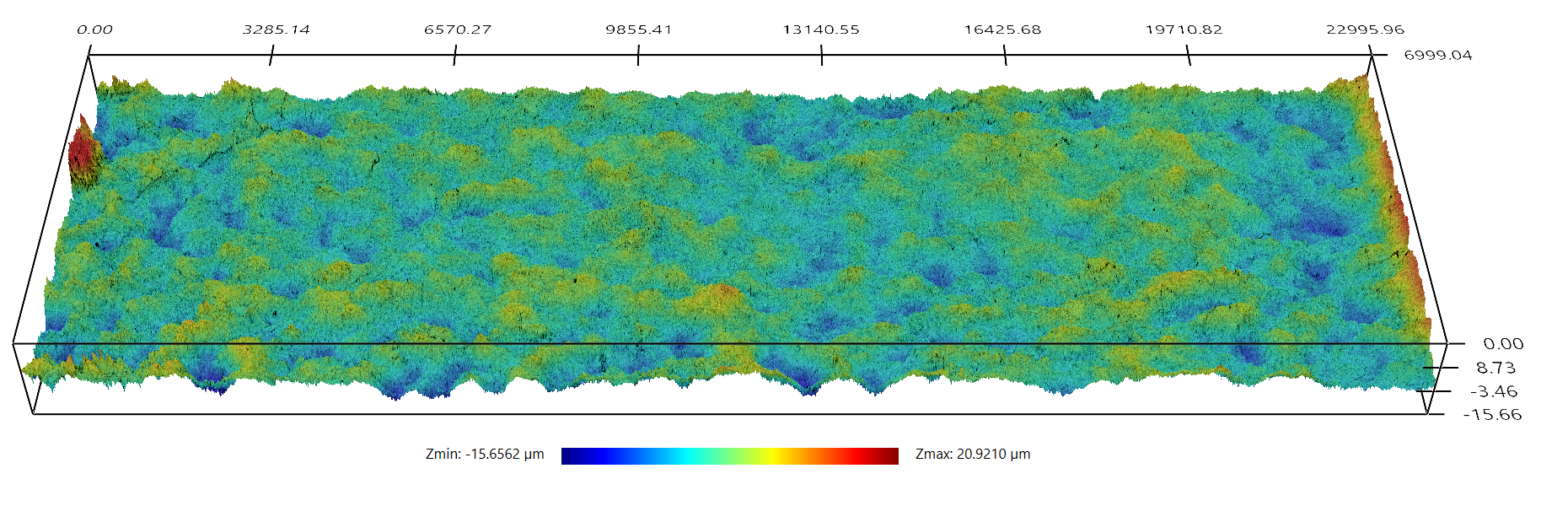

Supplement: Supplementary file 1 [file nanomaterials-14-01499-s001.zip › Tribology/profilograms/basic/fraction_over500_density1_3.png]

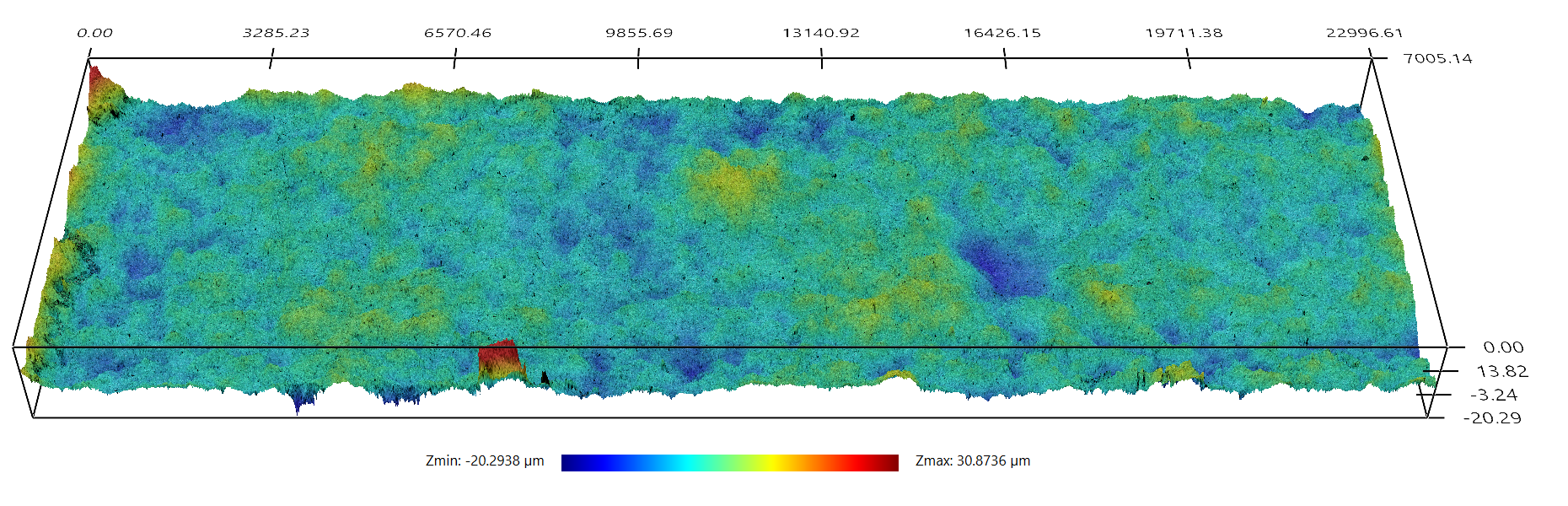

Supplement: Supplementary file 1 [file nanomaterials-14-01499-s001.zip › Tribology/profilograms/basic/fraction_over500_density1_6.png]

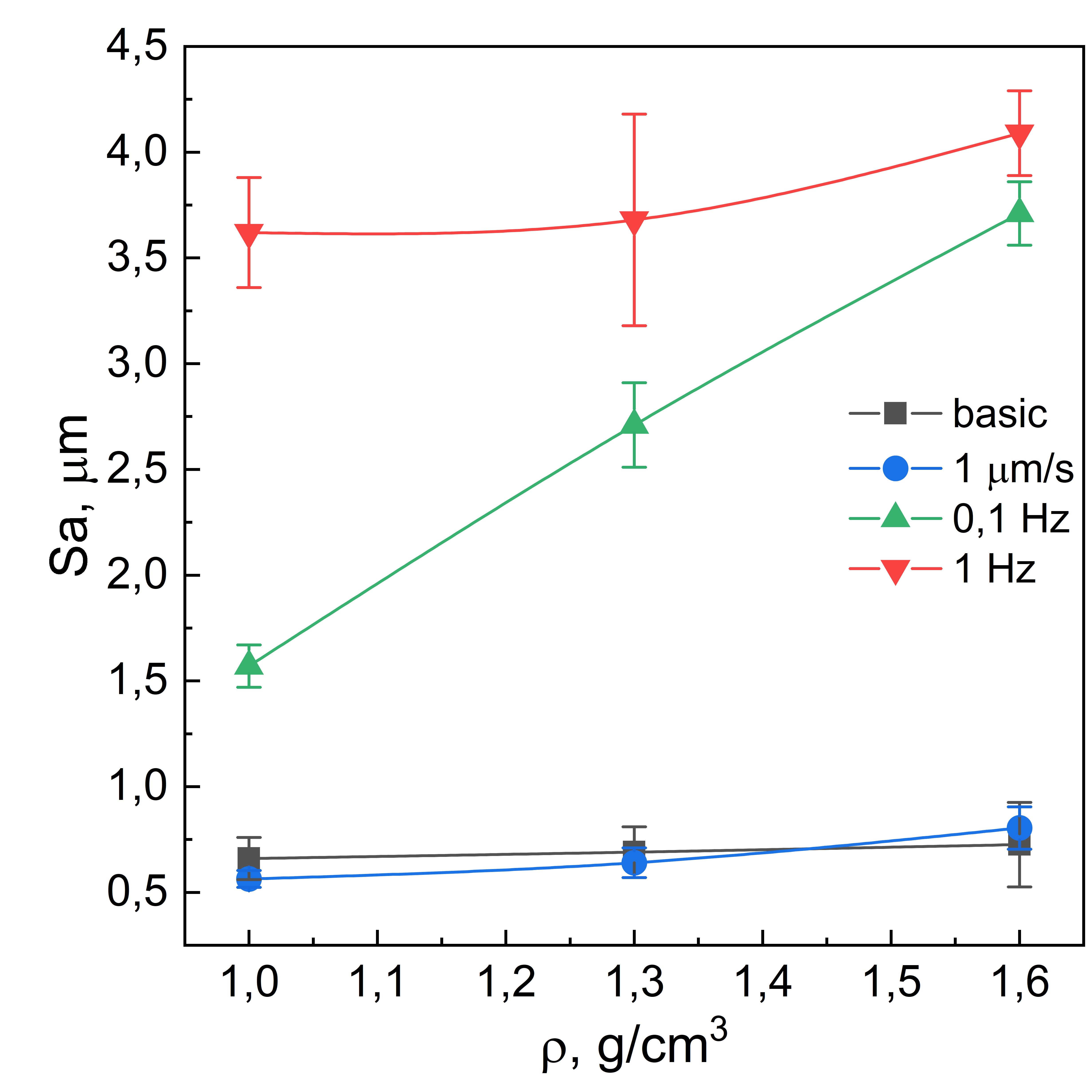

Supplement: Supplementary file 1 [file nanomaterials-14-01499-s001.zip › Tribology/roughness/Sa/160-200.png]

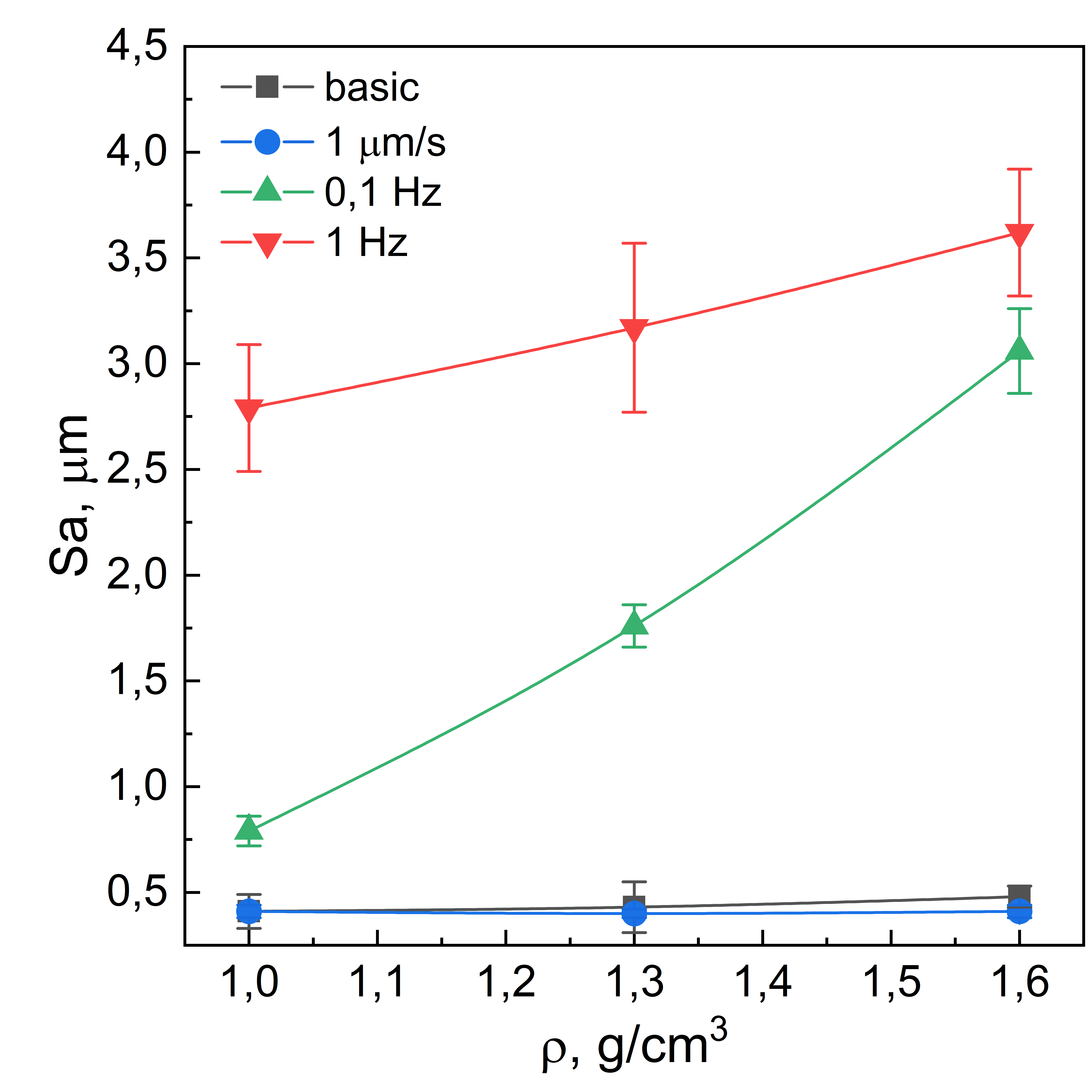

Supplement: Supplementary file 1 [file nanomaterials-14-01499-s001.zip › Tribology/roughness/Sa/40-80.png]

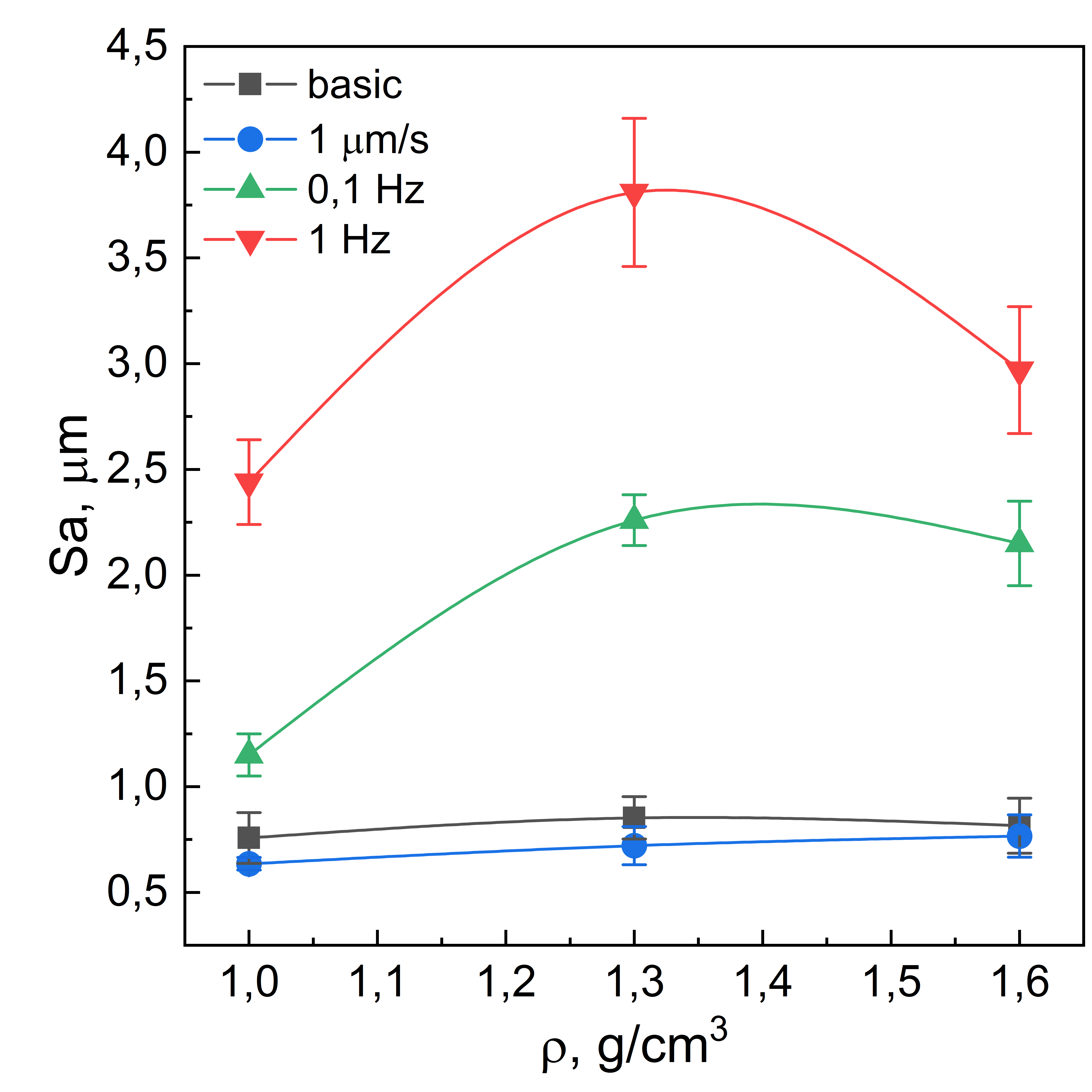

Supplement: Supplementary file 1 [file nanomaterials-14-01499-s001.zip › Tribology/roughness/Sa/over500.png]

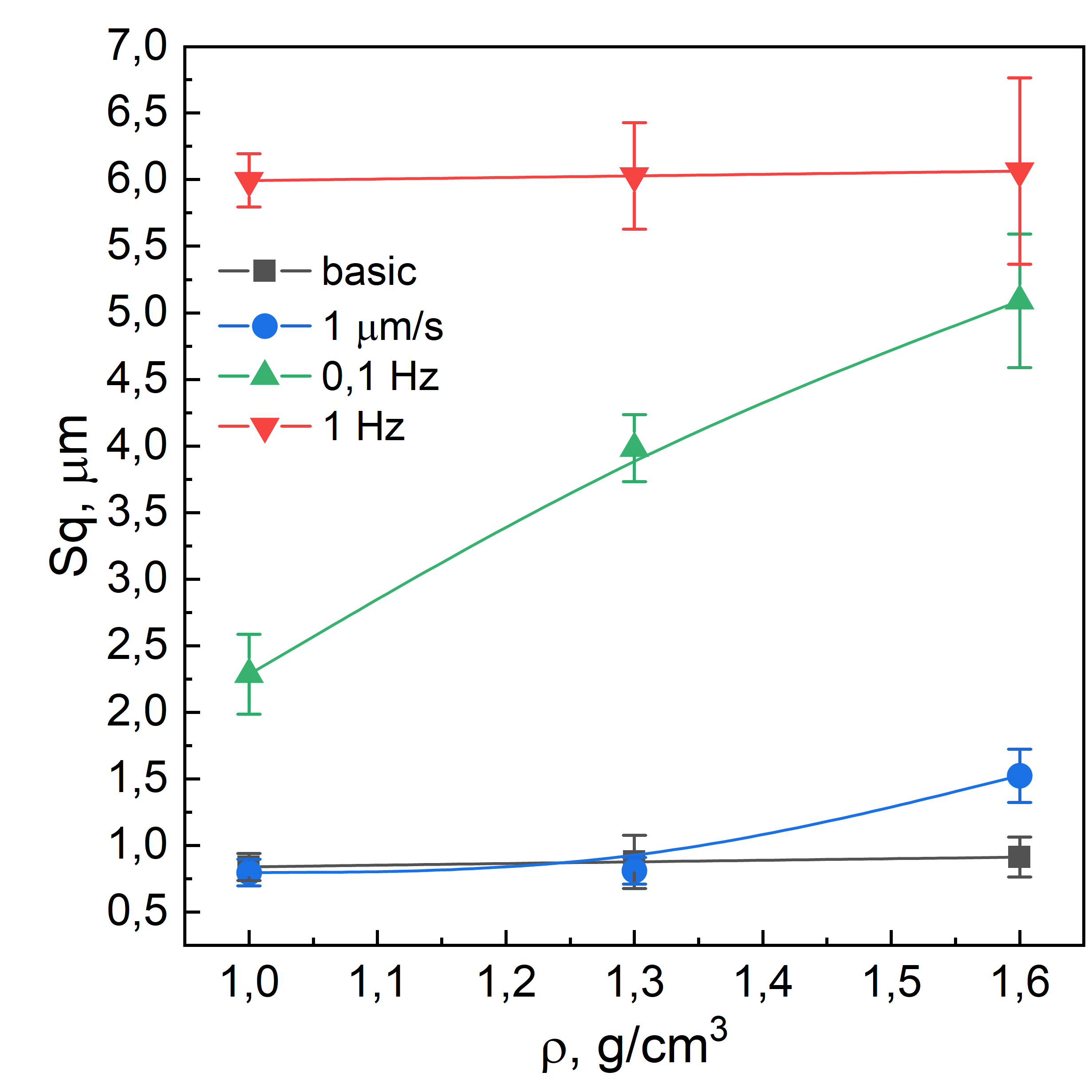

Supplement: Supplementary file 1 [file nanomaterials-14-01499-s001.zip › Tribology/roughness/Sq/160-200.png]

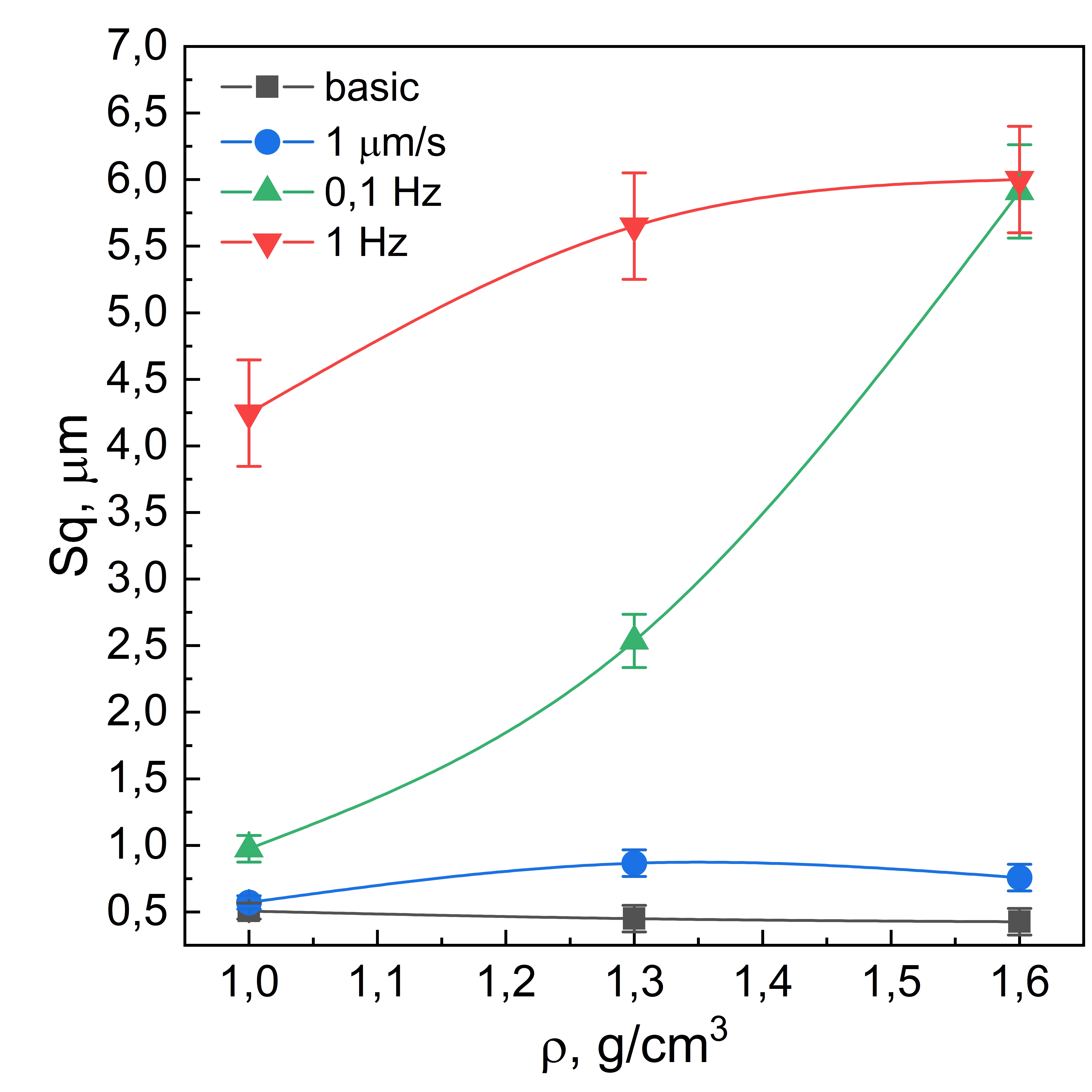

Supplement: Supplementary file 1 [file nanomaterials-14-01499-s001.zip › Tribology/roughness/Sq/40-80.png]

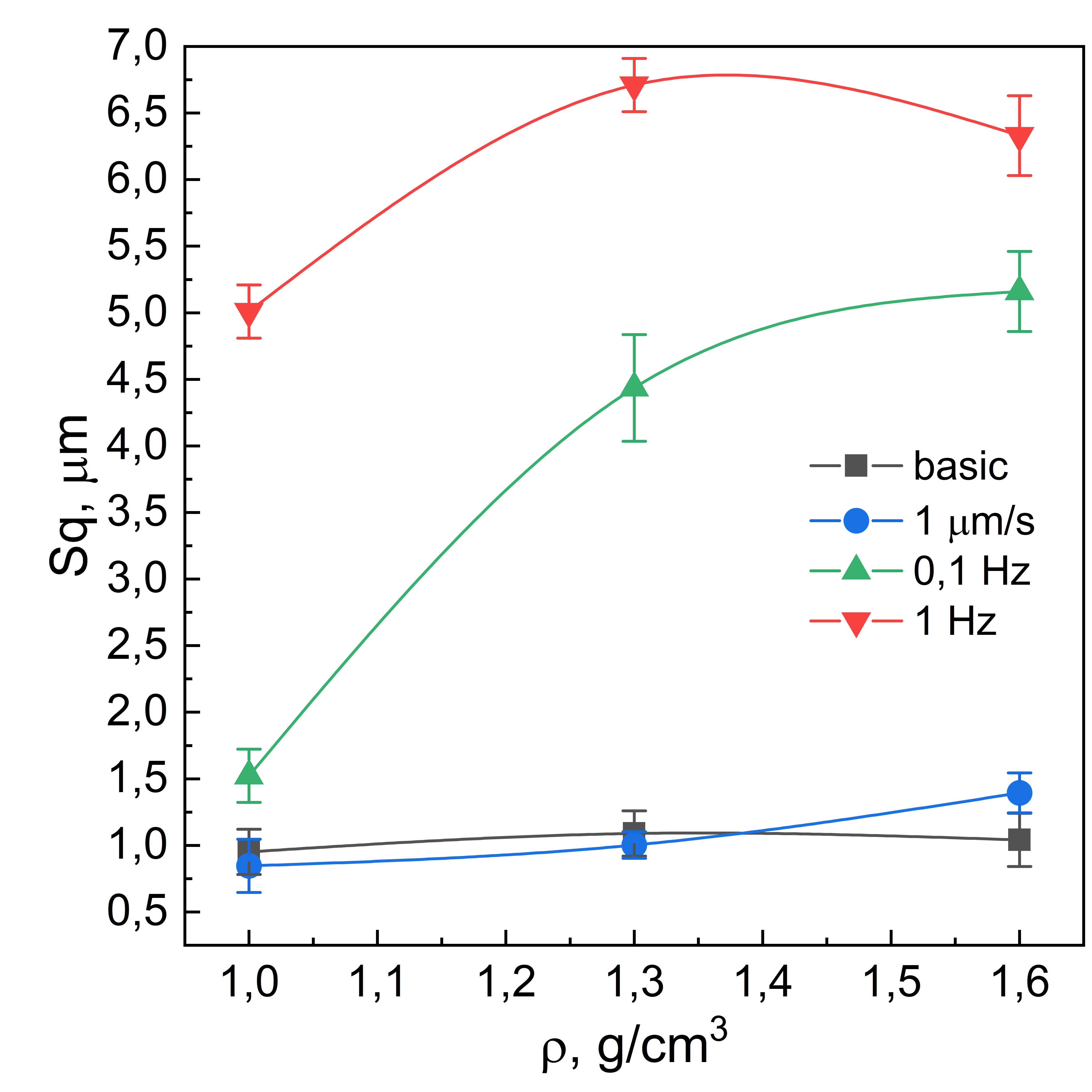

Supplement: Supplementary file 1 [file nanomaterials-14-01499-s001.zip › Tribology/roughness/Sq/over500.png]
